# Self‐Disassembling Macroporous Metal–Organic Framework‐Based Micromotors with Magnetically Controlled Motion for Sequential Drug Release

**DOI:** 10.1002/smtd.202500724

**Published:** 2025-06-03

**Authors:** Javier Bujalance Fernández, Víctor de la Asunción‐Nadal, Beatriz Jurado Sánchez, Alberto Escarpa

**Affiliations:** ^1^ Department of Analytical Chemistry Physical Chemistry and Chemical Engineering Universidad de Alcala Alcala de Henares Madrid E‐28802 Spain; ^2^ Andrés M. Del Río Chemical Research Institute Alcala de Henares Madrid E‐28802 Spain

**Keywords:** 5‐fluorouracil, cancer cells, delivery, doxorubicin, zeolitic imidazole frameworks

## Abstract

Herein, the synthesis of macroporous zeolitic imidazole framework (ZIF)‐based magnetic micromotors (MMs) for the dual encapsulation of 5‐fluorouracil (5‐FU) and doxorubicin (DOX), with pH‐triggered release is described. The MMs are synthesized in methanolic solutions, using superparamagnetic iron oxide nanoparticles (Fe₃O₄) as seeding agents and magnetic engines. The macroporous structure facilitates the loading of high doses of 5‐FU, while DOX is docked on the surface of the MMs. This results in release rates of 72.8 µg mg MMs⁻¹ of 5‐FU and 3 µg mg MMs⁻¹ of DOX. The magnetic motion of the MMs enables targeted delivery to predefined locations, allowing interaction with cancer cells in the culture medium. Under acidic conditions, DOX becomes protonated and is released from the surface of the micromotors, demonstrating rapid‐release kinetics. Simultaneously, the ZIF‐8 structure degrades under these conditions, enabling the sustained release of 5‐FU for continuous treatment. This dual‐delivery, pH‐induced mechanism is demonstrated in vitro using Caco‐2 cells as a relevant biological model, revealing a decrease in viability and diffusion of the drugs released into the cells. This is the first magnetic metal–organic framework (MOF)‐based MM that is pH‐sensitive and capable of sequentially releasing two drugs.

## Graphical abstract

MOF‐based magnetic micromotors release two drugs in a controlled and sequential manner at the acidic cancer cell media.

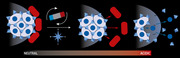

## 1 Introduction

Metal–Organic Frameworks (MOFs) are versatile materials composed of metal nodes linked to coordinated ligands in 3D, ordered structures and networks.^[^ ^1^ ^]^ Some MOFs exhibit rapid self‐degradation, along with exceptional inner surface areas and high biocompatibility, making them particularly attractive for biomedical applications.^[^ ^2^ ^]^ Indeed, MOF structures can be tailored to control their size, structure and loading capacity for drug delivery applications.^[^ ^3^ ^]^ Zeolitic imidazole framework (ZIF) MOFs are especially suitable for drug delivery due to their high biocompatibility and relatively low cytotoxicity. In particular, ZIF‐8 MOFs exhibit a coordination bond between the metal node and the ligand that breaks down at pH values between 1 and 6. While this may be a disadvantage in certain cases, it is an ideal feature for controlled drug delivery and pH‐induced degradation in cancerous environments, which are more acidic than healthy tissues.^[^ ^4^ ^]^

Micromotors (MMs) are microscale devices capable of autonomous propulsion in solution.^[^ ^5^ ^]^ The combination of MOFs with MMs offers an ideal platform for simultaneous drug encapsulation and dynamically controlled delivery to specific areas. Early designs in this area have relied on (bio)catalytic propulsion, using porous ZIF‐67 MOF‐based MMs with a cobalt metal core for DOX encapsulation,^[^ ^6^ ^]^ as well as ZIF‐L/catalase/DOX^[^ ^7^ ^]^ and ZIF‐L/catalase/5‐FU‐loaded MMs. While pH buoyancy control has been demonstrated in MCF‐7 cells, the necessary use of peroxide as a fuel restricts its applicability in cellular settings, in addition to potential enzyme deactivation and media interference with propulsion.^[^ ^8^ ^]^ Among biocompatible propulsion methods, magnetic fields are ideal for biomedical applications, considering their high biocompatibility and potential use with clinical Magnetic Resonance Imaging (MRI) systems.^[^ ^9^ ^]^

Magnetic actuation of MMs can be classified into two main categories: i) rotating or oscillating magnetic fields, and ii) magnetic gradients. Rotating and oscillating fields generate torque‐driven motion, while magnetic gradients impose force‐driven motion. To actuate MMs using rotating or oscillating magnetic fields, the structural features of the MMs are employed to apply translation in the desired direction. For example, helical structures can be propelled in a corkscrew motion along their long axis under a rotating magnetic field,^[^ ^10^ ^]^ while linear structures with flexible hinges can perform “fish‐like” motion under oscillating magnetic fields.^[^ ^11^ ^]^ The speed of MMs under rotating/oscillating magnetic fields is primarily limited by the structural step‐out frequency, and these systems typically require more complex designs involving various electromagnets (e.g., Helmholtz coils to generate uniform magnetic fields or ferromagnetic core magnets to generate localized magnetic gradients), synchronization stages and advanced control systems. On the other hand, magnetic gradients can be applied to any magnetic structure, regardless of its geometry, and their design and operation tend to be simpler. However, this method does present some drawbacks. First, the intensity of magnetic gradients decreases with the square of the distance, making the system highly dependent on the relative positioning of the objects. Second, to experience magnetic thrust, objects must exceed a threshold magnetic field, especially at low Reynolds numbers where inertial forces are negligible. Pane's group made an important contribution in this area by reporting the motion of ZIF‐8/Ni/Ti/polydopamine helical swimmers, which were propelled in a corkscrew motion within defined patterns by rotating magnetic fields generated by three pairs of Helmholtz coils.^[^ ^10^ ^]^ This concept was later extended using gelatine methacryloyl gel‐based materials to construct helical chassis for the assembly of DOX‐loaded ZIF‐8@Fe particles, resulting in fully biodegradable MMs.^[^ ^12^ ^]^ Mesoporous ZIF‐8@Fe MMs loaded with DOX have been used as cooperative swarming units for controlled delivery and pH‐triggered release (pH 5.5–6.5) in the T24 in vivo model.^[^ ^13^ ^]^

Inspired by the compatibility of magnetic MOF‐based MMs with biological systems (in terms of propulsion and materials), we describe the synthesis of macroporous ZIF‐8‐based MMs with an internal Fe₃O₄ core (ZIF‐8@Fe₃O₄), enhancing the inner surface area for drug encapsulation and enlarging the pore size for improved drug loading. The magnetic ZIF‐8 MMs are propelled by the combined action of rotating fields and magnetic gradients. A custom‐built electromagnetic system was designed to impart a rotating motion to the MMs as a “magnetic kick‐starter” to overcome viscous drag. The rotating field is applied by rotating two permanent magnets, simplifying the required hardware and software design. Thanks to the mechanical advantage provided by this local rotating motion, magnetic propulsion can be realized at much lower magnetic field intensities. For the first time, the controlled encapsulation of two different drugs with different molecular sizes, namely 5‐fluorouracil and doxorubicin, into the ZIF‐8@Fe₃O₄ MMs is thoroughly evaluated. The magnetic nature of the MMs, combined with the computer‐controlled movement of the set‐up, enables controlled drug delivery to the targeted site and selective release of antitumor drugs via pH change, as demonstrated using Caco‐2 cancer cells as a model. The unique structural features of MOFs allow for controlled drug distribution, with 5‐FU encapsulated within the porous MOF structure and DOX docked on the surface, enabling sequential drug delivery. The combination of controlled motion and selective release allows the MMs to be directed through a cell culture to cancer cells, where they proceed to release their drug payload in response to the decreasing pH in the acidic environment.

## 2 Results and Discussion

### 2.1 ZIF‐8 Magnetic Micromotor Synthesis Rationale and Characterization for Controlled Dual‐Drug Encapsulation

**Figure 1:**
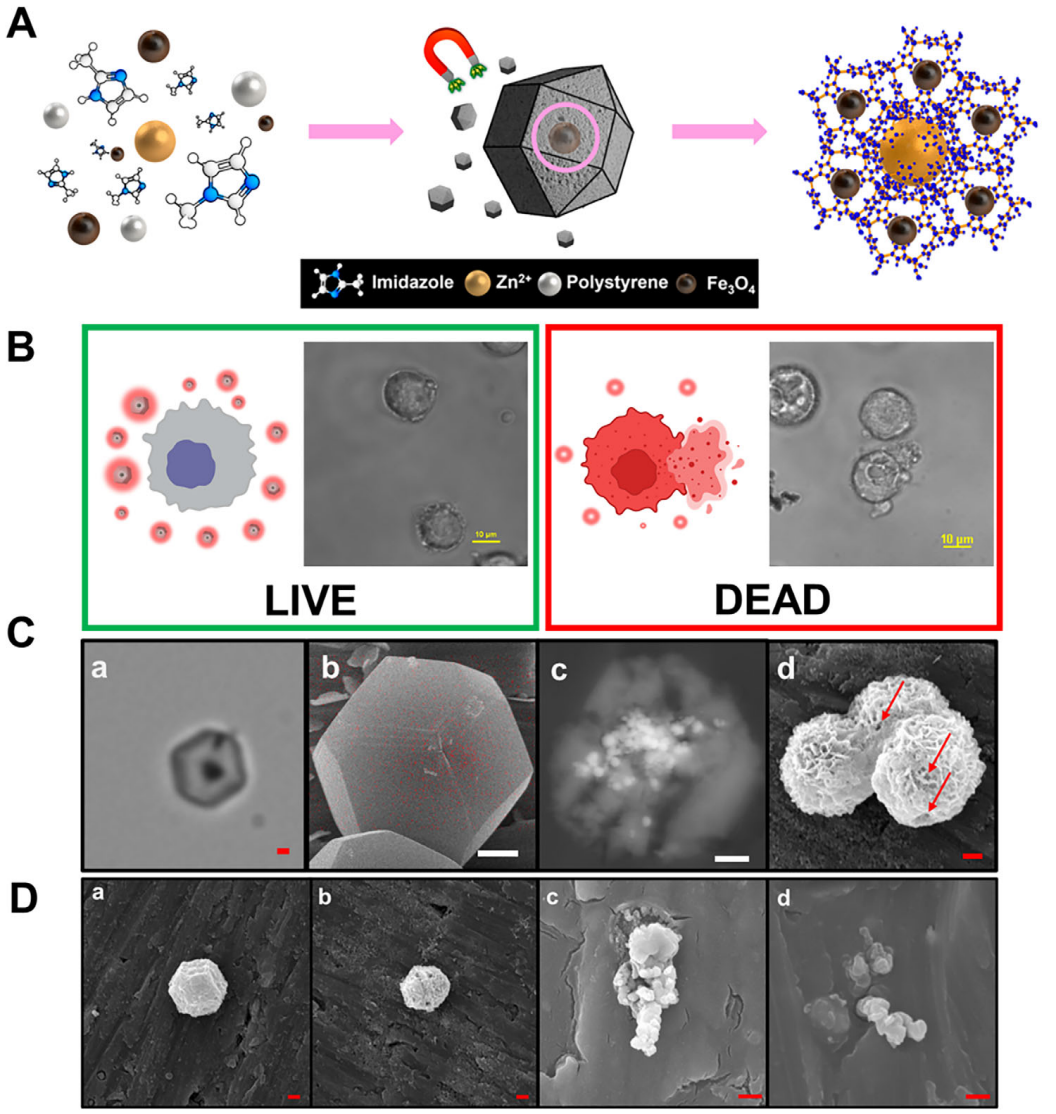
A) Schematic of the synthesis of magnetic macroporous ZIF‐8@Fe_3_O_4_ MMs. B) Schematic and time‐lapse microscopy images before (LIVE) and after (DEAD) ZIF‐8@Fe_3_O_4_ MMs rupture a Caco‐2 cell. Magnetic system conditions: electromagnet voltage = 5.5 V, rotating permanent magnet voltage = 1 V, clockwise direction. Scale bars: 10 µm. C) Images showing the morphology of macroporous ZIF‐8 with internal Fe_3_O_4_ taken by: a) optical microscopy, b) SEM and overlapping EDX mapping of Fe (red dots), c) BSE‐SEM of the transversal section and d) SEM images after treatment with H_2_SO_4_ (pH 4), showing some pores (red arrows). Scale bars: 1 µm. D) SEM images of the ZIF‐8@Fe_3_O_4_ MMs at: a) pH 5, b) pH 4 and MMs loaded with 5‐FU and DOX after (c) 1 h and d) 48 h at pH 4. Scale bars: 1 µm.

The successful synthesis of MMs is key for optimal drug encapsulation and magnetic propulsion features. The synthesis process of the MMs is illustrated in **Figure** **1**A, Figure S1 (Supporting Information) and the experimental section. To achieve a rhombic dodecahedron shape, a 4:1 ratio of methylimidazole precursors to Zn was employed.^[^ ^14^ ^]^ To increase the inner surface area of the ZIF units for accommodating the magnetic cores to enhance drug loading, we performed the synthesis of the MOFs in the presence of PS nanoparticles (100 nm) and Fe_3_O_4_ nanoparticles as nucleation points.^[^ ^15^, ^16^ ^]^ According to the literature, the surface of PS nanoparticles is negatively charged, which can induce the formation of nucleation sites for Zn^2+^, leading to subsequent ligand assembly and ZIF‐8 formation.^[^ ^17^ ^]^ Thus, both entities serve as nucleation points for ZIF‐8 formation, resulting in core–shell magnetic MMs with macroporous structures after dissolving the PS particles with organic solvents. We chose a hard‐templating strategy due to its reproducibility, ease of fabrication and the ability to customize the core size and core‐to‐shell ratio. PS is a commonly used hard template due to its solubility in most organic solvents, which simplifies the exploration of solvent mixtures compatible with the MOF structure while maintaining effective etching properties toward the PS cores. To the best of our knowledge, this technique consists of two main steps: 1) Synthesis of ZIF‐8 in the presence of PS microbeads to form core–shell ZIF‐8@PS structures, 2) Etching of the PS cores with dimethylformamide (DMF), leading to the dissolution of the PS cores and, 3) Washing of the resulting hollow or semi‐hollow structures (for further details, see the experimental section).^[^ ^18^ ^]^ This approach enables the encapsulation of a high load of simultaneous drugs with varying molecular sizes, while also facilitating controlled propulsion for targeted delivery to cancer cells. The synthesis is highly reproducible, yielding MMs with an average diameter/length of 2.8 ± 0.2 µm (*n* = 100, as measured by SEM), as reflected in the size distribution shown in Figure S2 (Supporting Information). The size is highly uniform, indicating that the synthesis process is highly reproducible, with uniform movement and drug release. Furthermore, a high yield of micromotors per batch is obtained, with an average of 20 million MMs/mL in each synthesis. Also, as illustrated in Figure 1B, the motion of the drug‐loaded MMs can be controlled by a tailored magnetic device, allowing for cell targeting, binding, rupture, and sequential drug delivery triggered by the self‐destruction of MMs in an acidic pH environment. This is further demonstrated in Video S1 (Supporting Information) and discussed below.

Prior to drug encapsulation, a comprehensive characterization of MMs was performed. As seen in the scanning electron microscopy (SEM) images of Figure 1C‐b, the MMs exhibit the typical rhombic dodecahedron structure of ZIF‐8 crystals,^[^ ^19^ ^]^ which facilitates their magnetic actuation and movement via stick‐slip interactions with the substrate.^[^ ^13^ ^]^ Red dots correspond to the energy‐dispersive X‐ray (EDX) map, which illustrates the distribution of Fe. The successful incorporation and morphology of the inner Fe_3_O_4_ nanoparticle core are observable in the optical and backscattered electron SEM (BSE‐SEM) images in Figure 1C–a,c. The structure of the ZIF‐8 crystals was further modified by immersing the MMs in DMF, which induced the formation of macropores due to the dissolution of the PS nanoparticles. This was followed by partial dissolution of the ZIF‐8 structure in H_2_SO_4_, revealing the macropores indicated by the red arrows (see Figure 1C–d). The X‐ray diffraction (XRD) spectra of ZIF‐8 and ZIF‐8@Fe_3_O_4_ MMs, compared with the theoretical XRD spectrum of ZIF‐8 (Figure S3, Supporting Information), reveal characteristic peaks at 2θ = 7.4° (011), 10.4° (002), 12.7° (112), 14.7° (022), 16.4° (013), 18.0° (222), 22.1° (114), 24.5° (233), 26.7° (134) and 29.6° (044).^[^ ^20^, ^21^ ^]^ Adsorption isotherms, performed using the micropore analysis mode (as the BET model did not fit our results),^[^ ^22^ ^]^ as can illustrated in Figure S4 (Supporting Information), show a reduction in micropore density, with a trend indicating the favoring of large pores. The volume and surface area are even lower after incorporation of Fe_3_O_4_ nanoparticles, suggesting that some inner space is occupied by the magnetic nanoparticles.^[^ ^23^ ^]^ Attenuated total reflectance infrared (ATR‐IR) spectroscopy characterization (Figure S5, Supporting Information) confirms the successful integration of PS particles into the macroporous structure. It is evident that PS particles are embedded within the ZIF‐8, as the maximum evanescent wave penetration depth aligns with the mean size of the ZIF‐8. Both morphological inspection (SEM) and chemical analysis (XRD, ATR‐IR) further demonstrate the successful removal of the templates, resulting in macroporous ZIF‐8 structures with high drug‐loading capabilities. To assess the ability of ZIF‐8@Fe_3_O_4_ MMs to encapsulate drugs and release them under pH‐triggered conditions in cancer cell environments, a degradation study was conducted by incubation at different pH values (Figure 1D). Minimal degradation was observed at pH 5. However, at pH 4, the MOF structure was compromised after 1 and 48 h, allowing for the slow release of encapsulated drugs. Given that pH values in the digestive tract can range from 4 to 5, Caco‐2 human colon adenocarcinoma cell lines were chosen as a model for further studies,^[^ ^24^ ^]^ making these MMs a promising platform for drug delivery in such environments.^[^ ^25^ ^]^

### 2.2 Magnetic Propulsion‐Guidance Capabilities: Principle, Design, Experimental Set‐Up and Trajectory Characterization

Following model selection, we optimized the propulsion and interaction of the ZIF‐8@Fe_3_O_4_ MMs with Caco‐2 cell cultures. In brief, our magnetic system consists of a 3D‐printed platform housing two permanent magnets and four electromagnets that generate switchable magnetic gradients. These gradients are controlled by an electric motor to generate rotating magnetic fields. The 3D‐printed device was custom‐designed to fit the stage of an inverted optical microscope, featuring four tilted cylindrical holders for the electromagnets, a holder for the permanent magnets and an opening to accommodate the microscope slide, as shown by the image in Figure S6A (Supporting Information). A full wiring schematic of the system is provided in Figure S6B (Supporting Information). The system is connected to a computer for image acquisition and motion control. In the experiments, the rotating field was fixed at a constant speed by maintaining a fixed applied voltage (1–1.5 V). Four electromagnets with ferromagnetic cores were employed, with a fixed voltage (0.5–10.5 V) controlled by relays that activated the corresponding electromagnets on demand. As shown in Figure S7A (Supporting Information), the apparent magnitude of the observed magnetic field remains constant, as it is primarily caused by the permanent magnets attached to the electromotor. The applied voltage only affects the rotational speed (Figure S7B, Supporting Information), with the RPMs increasing as the voltage increases. As seen in Figure S7C, Supporting Information), the apparent magnitude of the magnetic field is constant up to a threshold of ≈3 V. Beyond this threshold, the magnetic field increases linearly with the applied voltage, likely due to the initial voltage drop across the wire's resistance leading to low magnetic field intensity at low voltage.

From our experimental data, we hypothesize three main roles of the magnetic field: i) the magnetic field, in conjunction with a magnetic gradient, breaks the symmetry of the system, leading to non‐reciprocal actuation; ii) the local rotation induces shear flow around the particles, reducing hydrodynamic drag; and iii) the interactions between the particles and the substrate are minimized. Our system includes two distinct magnetic inputs: a rotating magnetic field and a magnetic gradient. The motion can then be characterized in three different scenarios, as observed in Video S2 (Supporting Information). The following cases were studied:

*Case 1. Magnetic gradient only*: When subjected to a magnetic gradient, a magnetic force is exerted on the particles, resulting in the alignment of the dipole moment (m) with the magnetic field (B_g_).

(1) $$F=\nabla \left(m\;\cdot \;Bg\right)$$

In this case, unless very strong magnetic fields are applied, the force becomes quasi‐symmetric in all directions, leading to a state of zero net displacement.

*Case 2. Rotating magnetic field only*: When rotating magnetic fields are applied, the transient magnetic field induces a torque on the nanoparticles.

(2) $$\tau =m\;\times \;B_{r}\left(t\right)$$

In the case of a homogeneous magnetic field, this generates rotation, imposing an angular velocity on the particles that are related to the frequency of the applied magnetic field (provided the frequency is lower than the step‐out frequency of the specific structure).

*Case 3. Rotating field and magnetic gradient*: In this case, the particles are simultaneously subjected to both rotation and a magnetic gradient, and the combined effects lead to a non‐zero net displacement of the nanoparticles. Under these conditions, the applied rotating magnetic field acts while the gradient establishes a preferential direction, which results in nonreciprocal motion, as the averaging over a full rotation no longer cancels out the forces. We can confirm that the imposed gradient breaks the symmetry, leading to nonreciprocal motion. The interactions with the surrounding medium are also influenced, particularly at low Reynolds numbers, where viscous forces dominate over inertia. In this situation, the rotation induces thinning of the boundary layer, which translates into reduced hydrodynamic drag. Furthermore, the rotating magnetic field minimizes the contact between the particles and the substrate, thereby reducing adhesive interactions. In conclusion, these results confirm that magnetic gradients can orient the particles but do not induce translation. Rotating fields induce localized rotation without translation, and translation is only observed when both a rotating field and a magnetic gradient are applied simultaneously.

We next tested the influence of the magnetic set‐up and the capacity of the MMs for controlled motion in cellular media. Videos were recorded using a concentration of 500 000 MMs mL^−1^. The MM solutions were sonicated before recording the videos to prevent the formation of clusters. The direction of the moving MMs was switched by activating or deactivating electromagnets (5.5 V) on demand to drive the MMs to the desired location. During this process, the rotating permanent magnets remained active at 1 V to facilitate the movement of the MMs.

**Figure 2:**
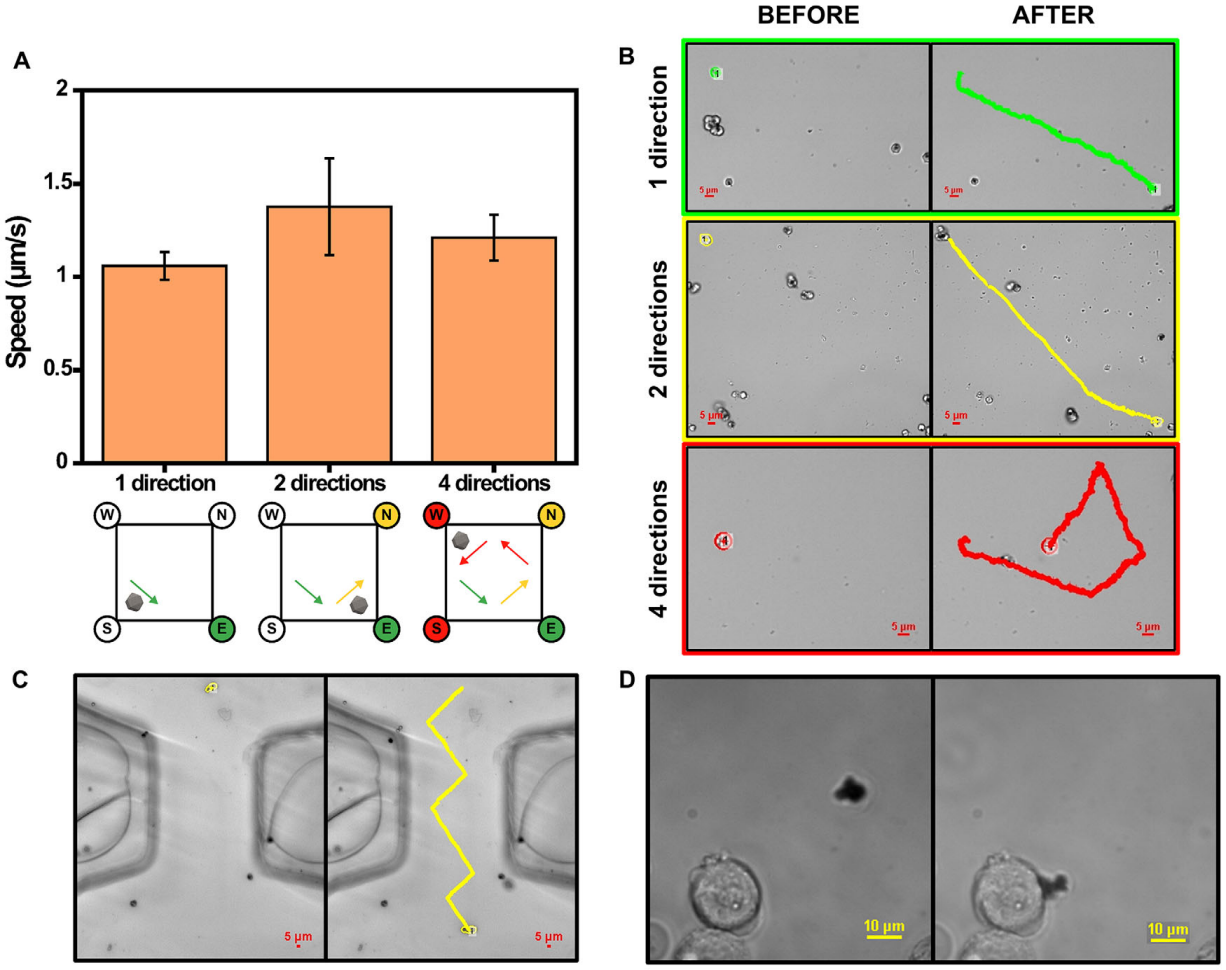
Optimization of the magnetic propulsion of macroporous ZIF‐8@Fe_3_O_4_ MMs. A) Speed measurements of magnetic macroporous ZIF‐8@Fe_3_O_4_ MMs in 1, 2 and 4 directions. Measurements were performed after electromagnetic actuation of “E” for 1 direction, sequential actuation of “E → N” for 2 directions, and sequential actuation of “E → N → W → S” for 4 directions. In all cases, the rotating permanent magnet was active, and only one electromagnet was activated at a time. Each time the direction changed, one electromagnet was switched off and another was immediately switched on. “E, N, W and S” represent the different electromagnets. Error bars represent the mean values ± standard deviation (*n* = 5). B) Time‐lapse images (taken from Video S4, Supporting Information) of ZIF‐8@Fe_3_O_4_ MMs before and after activation of the magnetic system, in 1, 2 and 4 directions. C) Time‐lapse images (taken from Video S5, Supporting Information) of ZIF‐8@Fe_3_O_4_ MMs moving through a PDMS channel (width = 140 µm) with the electromagnet system. D) Time‐lapse microscopy images (taken from Video S6, Supporting Information) in which an MM is driven toward a Caco‐2 cell, attaches to it and shakes it. (Conditions: electromagnet voltage = 5.5 V, rotating permanent magnet voltage = 1 V and clockwise direction).

As shown in **Figure** **2**A, the linear speed of MMs remains similar, despite the change in the direction of the magnetic MMs. These results demonstrate that driving the MMs in different directions does not affect their propulsion capabilities. We also tested the motion of the MMs in different biological media, such as simulated gastric acid (as the targeted application is aimed at the treatment of gastric tumors) and serum, as relevant biological media that can hinder the motion of the MMs. As observed in Video S3 and Figure S8 (Supporting Information), efficient motion is seen in serum, with linear speeds of 4.1 ± 1.4 µm s^−1^. In contrast, under the highly acidic conditions of gastric acid (pH 1), the ZIF‐core dissolves instantly, and the observed motion corresponds to the iron core, with speeds of 0.9 ± 0.4 µm s^−1^. Nevertheless, optimal motion is observed in serum, with adequate speeds for successful drug delivery. The relatively high speed observed can be attributed to a potential surfactant‐like effect of the serum components, which could reduce the surface tension and thereby increase speeds. This effect has been previously observed in catalytic MMs.^[^ ^26^ ^]^ Additionally, as observed in the Video, no aggregation is noted in the serum samples. For future drug delivery applications, we tested the stability and potential aggregation of the MMs in acetate buffer, gastric acid and serum, as this can affect the release profiles. As shown in Figure S8a,b (Supporting Information), after 24 h in each medium, the ZIF structure dissolves efficiently, with some residues and magnetic nanoparticles present, but no apparent aggregation.

One of the most intriguing features of the developed MMs is their ability to be transported to targeted locations, as illustrated in the time‐lapse images of Figure 2B and corresponding Video S4 (Supporting Information). The MMs can be directed in four directions in the horizontal plane and effectively manoeuvred to targeted locations. It is also possible to redirect them through the channels of a microfluidic system by controlling both the direction and rotation inside the channel (see Figure 2C; Video S5, Supporting Information). To demonstrate the swimming capabilities in a biological media, 1 µL of the solution was dropped onto a well containing Caco‐2 cells, and optical microscopy videos were recorded while inducing the magnetic operation of the MMs. Successful motion of the MMs in culture cell media was achieved, as shown in Figure 2D and Video S6 (Supporting Information), illustrating the docking of a ZIF‐8@Fe_3_O_4_ MM cluster with a Caco‐2 cell. After contact with the cells, the interaction of the MMs with the cell membrane stops the translation. However, the rotating field can still actuate the MMs by inducing the rotation of the MMs on the cell surface. As observed, the motion of the MMs in the cell culture media toward Caco‐2 cells can reach speeds of up to 2.2 µm s^−1^. The coupling of ZIF‐8@Fe_3_O_4_ MMs to the cell can be achieved efficiently, and a rotating motion can be induced on the cell surface with adjustable speed (up to 3.3 Hz), potentially causing cell lysis if required.

### 2.3 Drug Encapsulation in the MMs and Release Profiles

**Figure 3:**
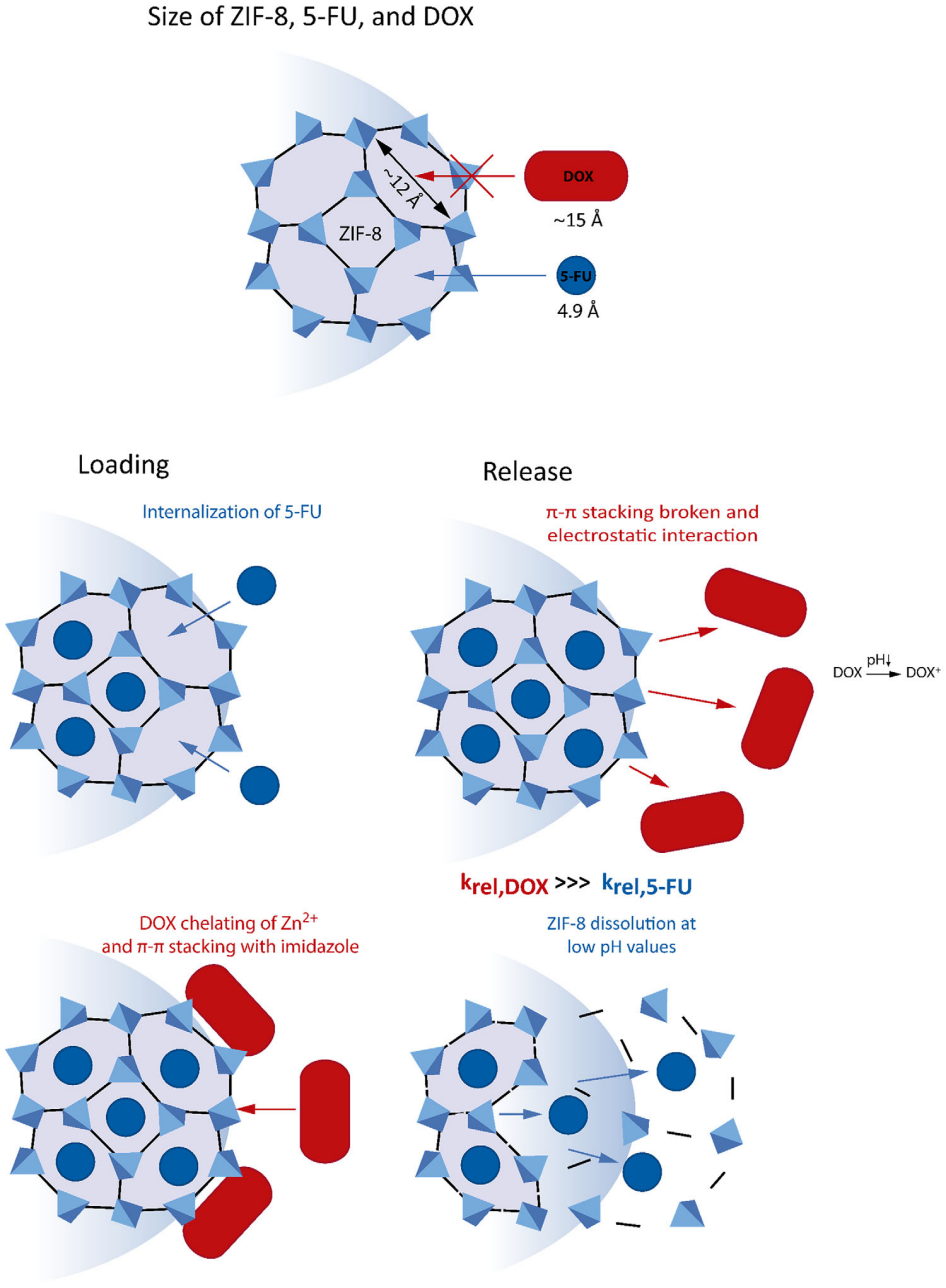
Schematic of the main processes involved in the drug loading and release of both DOX and 5‐FU drugs on ZIF‐8 MMs (the size representation of ZIF‐8, DOX, and 5‐FU are not to scale). *k* = released kinetic constant.

ZIF‐8 exhibits a well‐defined porous structure with large pores (11.6 Å) that can encapsulate small therapeutic agents such as 5‐FU (average diameter 4.9 Å). However, due to the size of 5‐FU, leaching is observed even in the absence of noticeable degradation of the ZIF‐8 structure's integrity, due to the lack of specific interactions with the host structure.^[^ ^27^ ^]^ In contrast, DOX is a significantly larger molecule (15 Å) and, due to its size, cannot be internalized into the ZIF‐8 structure. Instead, DOX is known to interact with the Zn^2+^ cations via chelating sites on the anthracycline aromatic moiety,^[^ ^28^ ^]^ while π–π interactions with the imidazole ligands are also possible. Under acidic conditions, DOX becomes positively charged as the slightly basic amino group becomes protonated (pKa_NH2_ = 8.3).^[^ ^29^ ^]^ To summarize, the following processes occur during the loading and release of 5‐FU and DOX into ZIF‐8 MMs. Loading: i) Due to its small size, 5‐FU is internalized into the microporous structure of the MOF, ii) DOX is docked on the surface of ZIF‐8, blocking the pores and preventing the leaching of 5‐FU in basic and neutral environments, thus limiting the loading of 5‐FU to the desired therapeutic concentration. Release: i) at physiological pH, DOX remains unprotonated, but when the pH is altered to acidic conditions, DOX becomes cationic and is released into the media due to electrostatic interactions with the Zn^2+^ centers, along with the breaking of the π–π interactions.^[^ ^30^ ^]^ ii) From pH 6 to acidic media, the integrity of the porous ZIF‐8 structure is compromised, leading to the redissolution of the encapsulated 5‐FU. The processes involved in the encapsulation and delivery of 5‐FU and DOX in the ZIF‐8 structure are schematized in **Figure** **3** for clarity.

**Figure 4:**
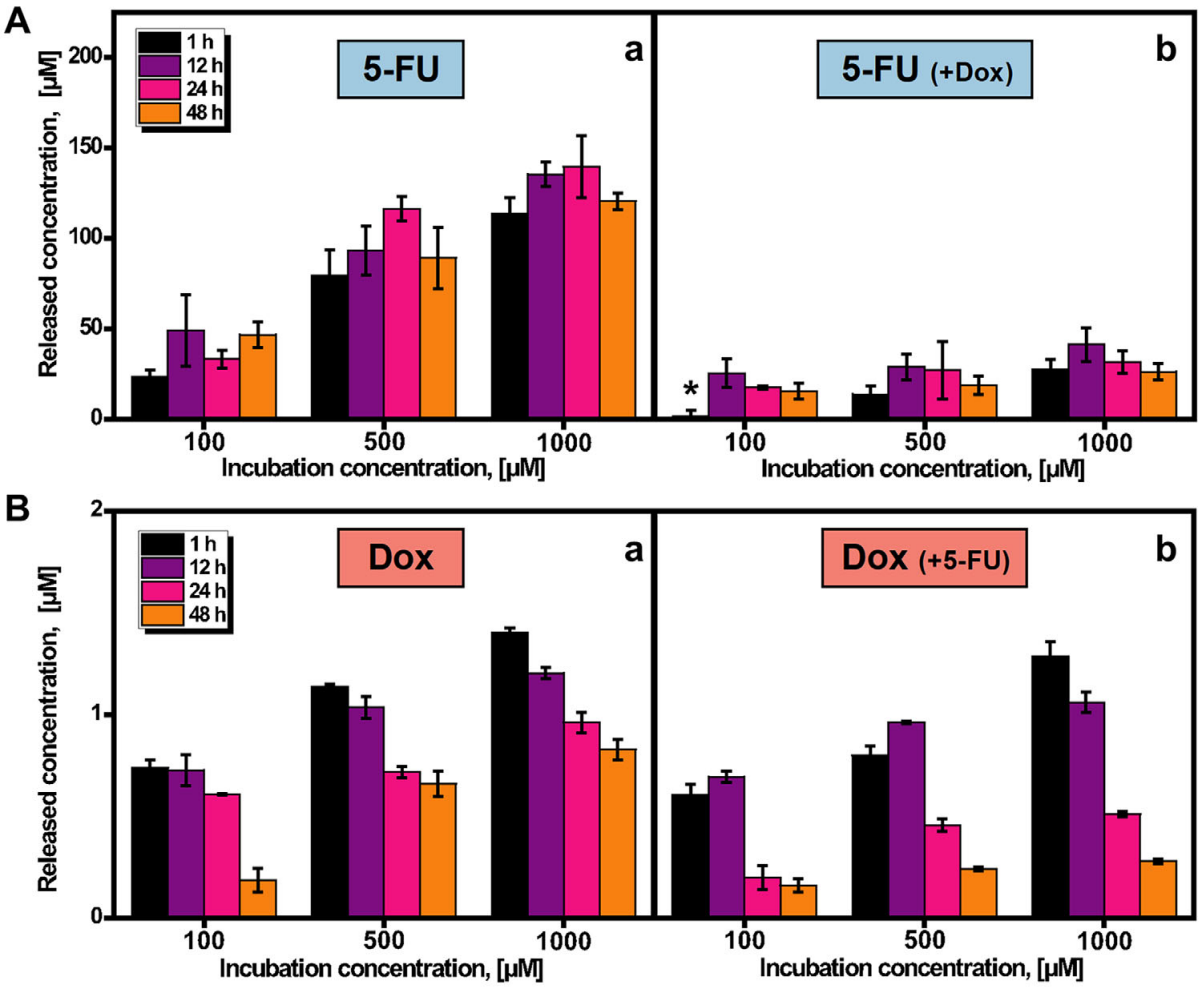
Drug release (in terms of released concentration) into the magnetic macroporous ZIF‐8@Fe_3_O_4_ MMs at pH 4, previously incubated with 3 different concentrations of: A) 5‐FU and the same concentration of 5‐FU in the presence of DOX. B) DOX and the same concentration of DOX in the presence of 5‐FU. * (<LOD). Error bars represent the mean values ± standard deviation (*n* = 3).

Once we characterized the successful motion and operation of the ZIF‐8@Fe_3_O_4_ MMs in biological media and examined the principles behind drug delivery from this MOF, we tested DOX and 5‐FU as model cargoes to demonstrate the feasibility of our microporous design. To this end, the MMs were incubated with three different concentrations of each drug, both separately and simultaneously (100, 500 and 1000 µm). For further details on the encapsulation process, please refer to the experimental section. To calculate the released concentration, standard curves were measured using aqueous solutions of 5‐FU or DOX (see Figure S9, Supporting Information). The corresponding calibration curves are: y = 7x + 11 for 5‐FU (r = 0.98; LOD = 5 µm) and y = 838x–0.2 for DOX (r = 0.999; LOD = 0.0007 µm). As shown in **Figure** **4**, drug release was measured at 1, 12, 24, and 48 h with magnetic actuation of MMs. Different release profiles were observed when the drugs were encapsulated individually or simultaneously. In the case of 5‐FU, the maximum release (140 µm) was observed after 24 h using 1000 µm of the drug, as the incubation steps were performed in 2 000 000 MMs, while the release experiments were conducted with 500 000 MMs, which did not affect cell viability (see Figure 6C‐a). This corresponds to a drug release efficiency of 56% (560 µm) (Figure 4A). However, when 5‐FU is encapsulated in the presence of DOX, the maximum release is reduced to 41 µm, and the drug release efficiency of 5‐FU decreases significantly to 16.4% (164 µM) after 12 h (see Figure 4A). In the case of DOX, generally, lower concentrations are encapsulated compared to 5‐FU. As seen in Figure 4B, the maximum release (1.4 µm) occurs after just 1 h (1000 µm concentration of DOX for incubation), with a drug release efficiency of 0.56% (5.6 µm). However, similar behavior is observed when incubating with 5‐FU at the same time, with a total release of 1.3 µm and a drug release efficiency of 0.52% (5.2 µm) (see Figure 4B). It can be concluded that a release of up to 72.8 µg of 5‐FU and 3 µg of DOX per mg MMs can be achieved. The underlying reason for the different release efficiencies lies in the different charges of each drug, which plays an important role in the interaction with ZIF8 MMs, along with the molecular size. To demonstrate that the macroporous structure allows for the high loading dose of 5‐FU, we prepared microporous ZIF‐8@Fe_3_O_4_ MMs (for details, see the experimental section) and compared the loading ability with that of macroporous MMs. As shown in Figure S10 (Supporting Information), the normalized per cent release with the macroporous MMs is nearly twice as high as compared with the microporous MMs, further supporting the larger inner surface area and higher loading capacity of the macroporous structures.

As discussed previously, 5‐FU is internalized into the porous structure of ZIF‐8, whereas DOX is loaded onto the surface. Modifications with PS particles and acidic pretreatment can render the surface of ZIF‐8 more porous, allowing more exposed surface area and higher DOX loadings. Indeed, the relative 5‐FU/DOX loadings can be tailored by modifying the microporous structure of the MMs to meet therapeutic needs. Similarly, the blocking of the porous ZIF‐8 structure by DOX can be used to limit the loading of 5‐FU into the MMs. If a moderate concentration of 5‐FU is required, loading can be performed simultaneously. On the other hand, for higher concentrations, both drugs can be loaded sequentially. As shown in Figure S10 (Supporting Information), sequential incubation with 5‐FU followed by DOX results in a 1.26‐fold higher loading than simultaneous incubation, because DOX does not prevent 5‐FU from entering the macropores. However, when the sequential incubation is reversed (DOX first, then 5‐FU), the loading drops to less than half, as DOX prevents 5‐FU from entering the macropores. In this case, due to the required dosage, the loading was performed simultaneously for simplicity, which was observed in both the macroporous and microporous structures.

### 2.4 In Vitro Drug Release with MMs

**Figure 5:**
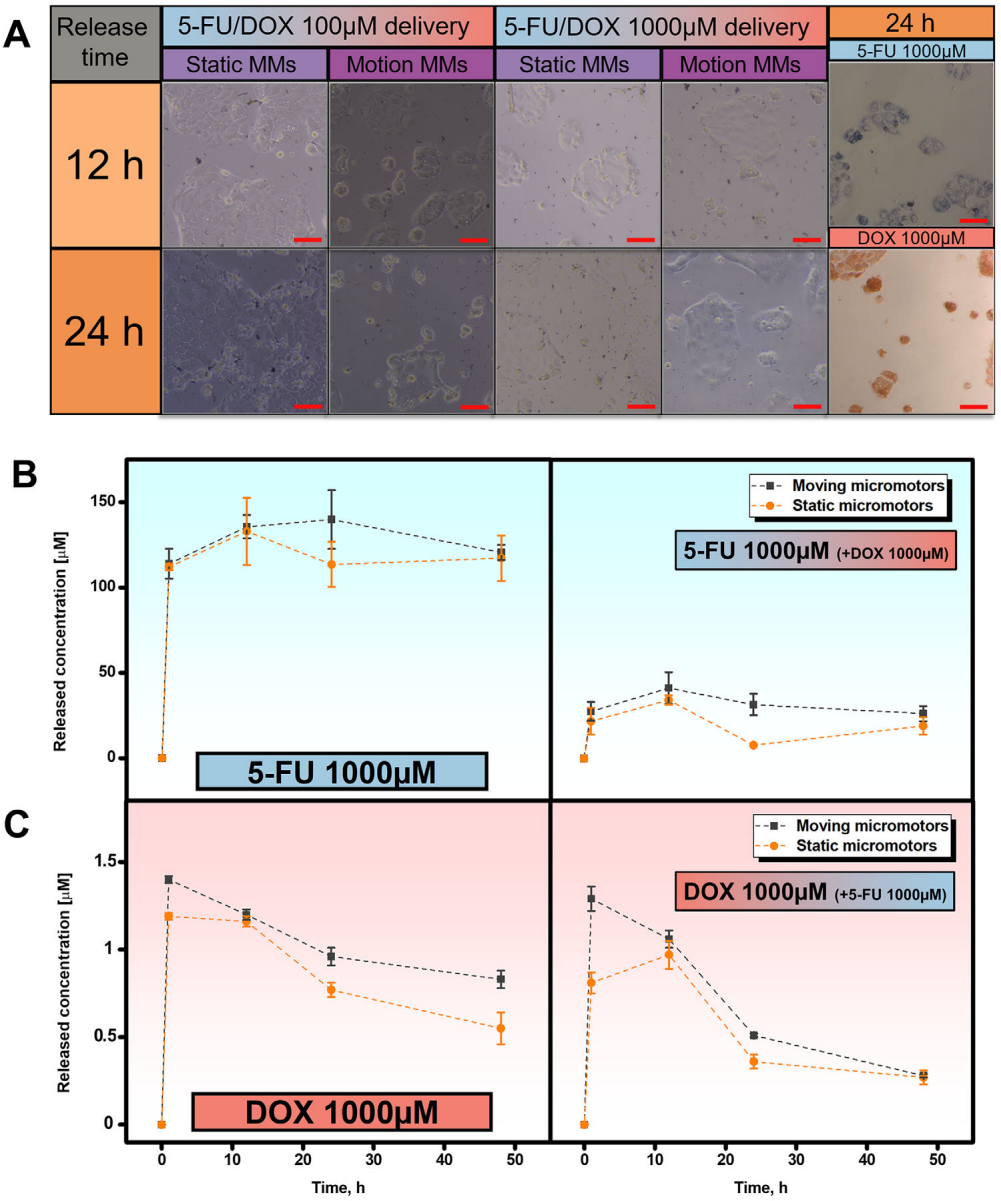
Drug release from the magnetic macroporous ZIF‐8@Fe_3_O_4_ MMs at pH 4. A) Confocal microscopy images of Caco‐2 cells after 12 or 24 h of incubation under static and magnetic conditions with MMs previously incubated with 100 or 1000 µm of 5‐FU and DOX simultaneously, compared with a solution of 1000 µm 5‐FU and 1000 µm DOX. B,C) Corresponding release profiles for each condition. Scale bars: 50 µm. Error bars represent the mean values ± standard deviation (*n* = 3).

For experiments involving Caco‐2 cells, ZIF‐8@Fe3O4 MMs loaded with 5‐FU and DOX were resuspended in a cell culture medium (DMEM supplemented with 10% FBS and antibiotic/antimitotic, containing 500 000 MMs) and adjusted to pH 4 using H_2_SO_4_ (≈0.1 mm). To initiate the drug delivery process, the cell culture medium was removed and replaced with a fresh culture medium supplemented with the drug‐loaded MMs. All conditions, except the control, were incubated at pH 4. Incubations were carried out at 37 °C with 5% CO_2_ in the dark for either 12 or 24 h, under either static conditions or with magnetic stirring of the MMs at 3.3 Hz. The resulting confocal microscopy images in **Figure** **5**A clearly demonstrate the high cytotoxic efficiency of cancer cells treated with moving MMs, as evidenced by a reduced density of Caco‐2 cells and a loss of their characteristic flat morphology. The cells adopt more spherical and irregular shapes, and their cytoplasm exhibits irregularities indicative of apoptosis. Additionally, a higher rate of cell deactivation is observed as the drug concentration for encapsulation increases. When the same experiment was performed using the drugs directly, rather than with the MMs, at a concentration of 1000 µm, cell death was significantly higher. The drug release profiles over time are shown in Figure 5B,C, with noticeable differences between 5‐FU and DOX. For 5‐FU, a rapid initial release is observed, followed by a plateau at 24 h, where the release remains constant. It is worth noting that the amount of drug released is slightly higher when using moving MMs due to enhanced fluid mixing, which also results in a more efficient cell killing, as illustrated in the confocal images of Figure 5A.

As previously mentioned, a significant reduction in the amount of drug loaded and released is observed when DOX is also present in the MMs. This is due to the docking of DOX on the surface of ZIF‐8, which blocks the pores of the framework. However, as will be explained later, the synergistic effect of both drugs enhances the killing efficiency. For DOX, an instantaneous release is observed, reaching its peak within 1 h, after which the release gradually decreases over time. As with 5‐FU, the release of DOX is greater with moving MMs, emphasizing the crucial role of propulsion in both enhancing delivery and targeting cancer sites. It should also be noted that additional collisions between the MMs and cancer cells due to magnetic‐induced rotation could contribute to the inactivation process by damaging the cell membranes and facilitating drug penetration. From the release profiles, two key observations can be made: DOX is released within minutes after reaching the target location for immediate cancer cell killing, whereas 5‐FU is released in a slow but continuous manner, providing prolonged treatment for up to 48 h.

**Figure 6:**
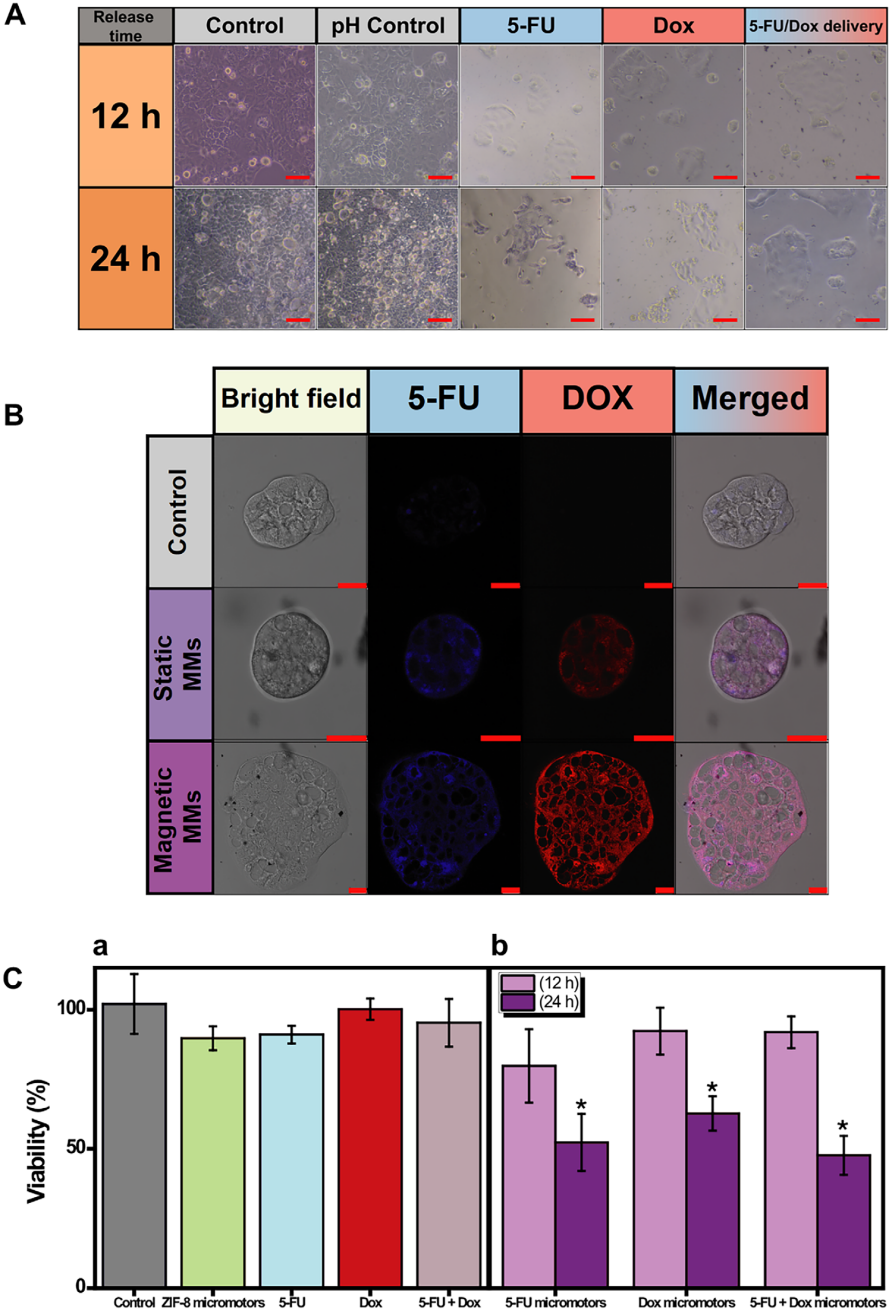
In vitro drug release study with the Caco‐2 cell model. A) Confocal microscopy images of Caco‐2 cells after 12 or 24 h of incubation under normal conditions (control), at pH 4 (pH control), treated with MMs at pH 4 previously incubated with 1000 µm 5‐FU, DOX, and both 5‐FU and DOX simultaneously. Scale bars: 50 µm. B) Confocal images of Caco‐2 cells after 24 h at pH 4 under static or magnetic conditions, with MMs previously incubated with 1000 µm of 5‐FU and 1000 µm of DOX simultaneously. 5‐FU was excited with a 405 nm laser, and DOX with a 561 nm laser. Scale bars: 25 µm. C) Corresponding MTT assays (*n* = 3). a) Conditions: pH 4 Control, unloaded MMs, 5‐FU (140 µm), DOX (1.4 µm), mix of 5‐FU (41 µm) and DOX (1.3 µm) under magnetic conditions after 24 h. b) Conditions: MMs in pH 4 previously incubated with 1000 µm of 5‐FU or DOX, or both, under magnetic conditions after 12 or 24 h. 500000 MMs/treatment. * Statistically significant difference between experimental and control conditions (α = 0.05). Error bars represent the mean values ± standard deviation (*n* = 3).

Once the release profiles were characterized, in vitro drug release studies were conducted using Caco‐2 cells as a representative gastrointestinal model over periods of 12 and 24 h. The experiments were carried out in a manner similar to the previous drug release profile studies, evaluating the role of MMs motion in the release process. Confocal microscopy images and corresponding MTT assays can be seen in **Figure** **6**, Figures S1–S13 (Supporting Information). Control experiments were also performed to assess any additional effects on cell apoptosis caused by improper cell preservation or the effect of sulfuric acid used to achieve the pH of 4 for rapid ZIF‐8@Fe_3_O_4_ dissolution and drug release. As shown by the integrity of the cells in Figure 6A and the 100% cell viability observed in the MTT assays, no adverse effects on cell viability were noted under both conditions. However, when moving MMs encapsulating DOX and 5‐FU, either individually or simultaneously, were used, a significant decrease in cell viability was observed, accompanied by visible cell apoptosis (see Figure 6A).

We further studied the potential diffusion and distribution of the drugs within the Caco‐2 cells using 5‐FU and DOX, which exhibit fluorescence properties that facilitate confocal microscopy observation.^[^ ^31^ ^]^ The results in Figure 6B show greater drug penetration under moving conditions compared to static conditions, with a notably densely packed area of DOX within the cells. Initially, the release was tested using H_2_SO_4_ to adjust the pH of the medium to the desired value. This method was used to optimize analytical detection and facilitate cell penetration observation. However, to more accurately simulate physiological conditions, release experiments were also conducted using acetate buffer to adjust the pH. Similar results (see Figure S11, Supporting Information) were obtained with sulfuric acid, with the only difference being a decrease in the observed fluorescence emission of 5‐FU in the merged image, which may be attributed to analytical limitations. It is also noteworthy that 5‐FU alone demonstrates lower cellular diffusion capability compared to when it is loaded in MMs together with DOX. However, the diffusion of DOX into the cell is similar whether administered alone or in combination with 5‐FU. In both cases, the release of the drugs from the MMs results in similar diffusion into the cells (see Figure S12, Supporting Information). In all conditions, it is evident that drug penetration into the cells is enhanced when MMs are actuated under magnetic conditions, likely due to the increased fluid microcurrents generated. This highlights the greater capacity for drug penetration under magnetic conditions. To gain further insight into this interaction, we captured SEM images of the cells after 24 h of contact and drug release from the MMs. Figure S14 (Supporting Information) shows intact cells with some MMs contacting the surface, along with masses or clusters of broken Caco‐2 cells mixed with aggregates of ferrite nanoparticles/undissolved ZIF‐8 MMs.

The results of the relevant MTT assays, using identical concentrations for both the MMs and the free drugs (Figure 6C–a show that the viability of Caco‐2 cells incubated with magnetically actuated ZIF‐8 MMs, 5‐FU, DOX, and a 5‐FU/DOX mixture remains above 90% after 24 h. In contrast (Figure 6C–b), statistically significant lower cell viability (≈45%) was observed after 24 h when using ZIF‐8@Fe_3_O_4_ MMs loaded with both DOX and 5‐FU simultaneously. Interestingly, cell death was similar when the drugs were released separately compared to when they were released together, likely due to the fact that the drug load is higher when the drugs are administered separately, despite the concentrations being lower when administered together. This suggests a synergistic effect, which is evident from the data in Figure S13 (Supporting Information). When the drugs were incubated separately in sequence, cell viability decreased to 32% when incubated first with 5‐FU and then with DOX (Figure S10, Supporting Information shows that the amounts loaded are higher), and increased to 64% when incubated first with DOX and then with 5‐FU (Figure S10, Supporting Information shows that the amounts loaded are lower), demonstrating the synergistic effect of the drugs. The action mechanisms of 5‐FU and DOX on cells differ; both kill cells via distinct pathways. This suggests that the method developed in this study can be used to administer various treatments with different concentrations, depending on the therapeutic requirements for each drug.

## 3 Conclusion

We have developed custom macroporous ZIF‐8‐based magnetic macroparticles (MMs) for the encapsulation of 5‐FU and DOX drugs, demonstrating relevant models and pH‐triggered release mechanisms. The magnetic propulsion of the MMs facilitates targeted delivery to predesignated locations, enabling interaction with cancer cells in the culture medium. This approach allows for controlled binding, transport, and membrane rupture. The macroporous structure enables high loading capacities. In comparison with previous studies (see Table S1, Supporting Information), this is the first instance of two drugs being encapsulated within MOF‐based MMs. Additionally, to the best of our knowledge, this is the first time a molecule has been employed both as a therapeutic agent and as a capping agent to limit the diffusion of a second therapeutic molecule. The release profile is significantly influenced by whether one or two drugs are encapsulated. A notable decrease in the loading and release of 5‐FU occurs when DOX is simultaneously encapsulated, as DOX docking on the surface of ZIF‐8 blocks the pores. However, the combination of two drugs within a single MM enables the rapid release of DOX for immediate cancer cell killing, alongside the sustained release of 5‐FU for continuous treatment. It is also important to highlight that in vitro results using Caco‐2 cells demonstrated a decrease in cell viability compared to treatments without MMs. The diffusion of the released drugs into the interior of the cells is enhanced by the potential generation of fluidic microcurrents induced by the magnetic movement of the MMs. The motility of the MMs, coupled with their biocompatibility and pH‐triggered degradation, presents considerable promise for the in vivo treatment of tumors in the near future.

## 4 Experimental Section

### Reagents and Materials

Methanol (cat. 34 860), 1‐Methylimidazole (cat. M50834), 2‐Methylimidazole (cat. M50850), Zinc nitrate hexahydrate (cat. 96 482), N, N‐Dimethylformamide (cat. 543 897), 100 nm polystyrene‐based microparticles (cat. 43 302), Dulbecco's Modified Eagle's Medium (DMEM) (cat. D6429), Foetal Bovine Serum (FBS) (cat. F7524), Human Serum (cat. H4522) and antibiotic Antimycotic Solution (cat. A5955) were purchased from Sigma‐Aldrich. Simulated gastric fluid (cat. 0 1651), Acetic acid (cat. 695 092) and Sodium acetate (cat. S2889) were purchased from Merck. Glass microscopic slides (cat. 12 302 158), Iron (II, III) oxide nanopowder (cat. 04 7141.36), 5‐Fluorouracil (5‐FU) (cat. 11 475 523), Sulfuric acid (cat. 12 656 777) and Whatman Cyclopore polycarbonate membranes (0.2 µm pores, 25 mm diameter; cat. 11 374 814) were purchased from Thermo Scientific (Spain). Protein LoBind tubes (1.5 mL; cat. 00 301 08116) were purchased from Eppendorf. Doxorubicin hydrochloride (cat. T1020) was purchased from Tebubio (Spain). µ‐Dish plates for confocal imaging (cat. 8115) were purchased from Inycom (Spain). Colon adenocarcinoma Caco‐2 cells (cat. ATCC‐HTB‐37; Lot No. 70 046 148) were purchased from LGC Group (England). Milli‐Q water was obtained using a Millipak Express filter (cat. MPGP04001) and Vent Filter (cat. TANKMPK01) from Merck Millipore. All reagents were used without further purification.

### Equipment

A DynaMag‐2 magnetic rack (cat. 12321D) was used to hold and wash the MMs. An ultrasonic bath (Elmasonic S 30 H; cat. 100 1955) and a probe CV18 attached to a Vibra‐Cell VCX 130 ultrasonic processor were employed for ZIF‐8 magnetization. A 3D printer (Original Prusa i3 MK3S+) was used to print the electromagnet system holder stage. An Eppendorf Centrifuge 5430, coupled with an FA‐45‐30‐11 rotor, was used for cleaning steps and material synthesis. Images and videos were captured with an inverted Nikon Eclipse Instrument Inc. Ti–S/L100 optical microscope, coupled with a Zyla sCMOS camera. The speed of the MMs was recorded and measured using NIS‐5.41 Elements software. A confocal microscope (Leica TCS SP5) equipped with He–Ne (561 nm) and UV (405 nm) lasers was used for fluorescence imaging of 5‐FU and DOX in cells. An optical microscope (Leica TCS‐SL) was employed to capture images of cultured cells. X‐ray diffraction (XRD) analysis was performed at the X‐ray Centre of the Complutense University. MMs were characterized by scanning electron microscopy (SEM) using a JSM 6335F (JEOL) with a secondary electron (SE), backscatter electron (BSE) and energy‐dispersive X‐ray (EDS) system (Xflash detector 4010, Bruker), as well as by transmission electron microscopy (TEM) (Zeiss EM10C). An Eppendorf ThermoMixer C was used as an incubator for the synthesis of macroporous ZIF‐8@Fe_3_O_4_ and drug loading. IKA KS 3000i Control was employed as an incubator for drug release experiments from ZIF‐8@Fe_3_O_4_ MMs. A Heschen electromagnet solenoid (P30/22, 30 mm, 12 V) was used as the magnetic field source, powered by a Stamos Soldering S‐LS‐75 Laboratory Power Supply. Fourier‐transform infrared (FTIR) analysis was carried out using a Nicolet iS20 (Thermo Scientific) to detect polystyrene residue in modified ZIF‐8. MPS method analysis was performed at the “*Unidad de Análisis de Sólidos Porosos*” of the University of Malaga.

### Evaluation of Penetration Depth in IR‐ATR Measurement

The penetration depth of the evanescent wave is defined by the general expression, assuming the refractive indices of diamond (nATR = 2.4) and ZIF‐8 (nsample = 1.38), an angle of incidence (θ = 40°), and a wavelength (λ = 17 µm). Under these conditions, the maximum penetration depth is ≈3 µm. This penetration depth is within the size range of the ZIF‐8 particles, meaning that the technique provides information about the outer shell of the nanoparticles, but not the core.

### Synthesis of Macroporous ZIF‐8@Fe_3_O_4_

For the synthesis, Zn(NO_3_)_2_·6H_2_O (50 mm) was dissolved in 50 mL of methanol by magnetic stirring. Separately, 1‐methylimidazole (200 mm) and 2‐methylimidazole (200 mm) were dissolved together in 50 mL of methanol using the same method. The two solutions were then mixed, followed by the addition of 10 µL of PS nanoparticles and 5 mg of Fe_3_O_4_ (resuspended in 1 mL of methanol, which had been sonicated for 5 min in an ultrasonic bath). The mixture was covered and incubated overnight at room temperature with shaking. All glassware and magnetic Teflon components used during the incubation were precleaned with aqua regia to prevent any external particles from acting as aggregation sites. After overnight incubation, an opaque supernatant formed. The supernatant was washed three times with methanol through polycarbonate membranes under a vacuum to remove residual iron oxide and PS nanoparticles from the solution. To remove the PS encapsulating the micromotors, the powder obtained in the previous step was resuspended in DMF, sonicated for 1 min in an ultrasonic bath, and incubated for 1 h at 700 rpm at 50 °C. This step was repeated twice. To estimate the quantity of micromotors (MMs) obtained per batch and achieve the desired concentration for each experiment, 1 µL of the MMs suspension was observed using a 4× objective, and then four additional measurements were made at 20× magnification at different locations within the drop. The average number of MMs per area was calculated by measuring the area of the 1 µL drop (using the 4× objective) and counting the number of MMs in each 20× image (with a known area). This method was repeated four times for each batch of MMs. Finally, the MMs were resuspended to a volume that provided the required concentration for the experiments.

### Synthesis of Microporous ZIF‐8@Fe_3_O_4_

The synthesis followed the same procedure as for the macroporous ZIF‐8@Fe_3_O_4_, except that PS nanoparticles and DMF incubation steps were omitted to prevent the formation of the macroporous internal structure.

### Electromagnetic System for Propulsion of MMs and Procedure for Video Recording

The setup consisted of four electromagnets and one electromotor with two attached permanent magnets. The electromagnets generated switchable magnetic gradients, while the rotating permanent magnets produced a rotating magnetic field. Arduino IDE 1.9 was used to develop a program to control the Arduino Nanoboard. The rotating field was fixed at a constant speed by maintaining a fixed applied voltage (1 V). For the magnetic gradient, four electromagnets with ferromagnetic cores (diameter 30 mm, length 22 mm) were used, with a fixed voltage of 5.5 V applied to four relays, which were activated on demand to supply voltage to the corresponding electromagnets. An Arduino Nano board was used to switch the relays, and the microcontroller's serial port served as the user interface to enter commands for the desired direction of the magnetic gradient. A stage to hold all magnetic elements was designed using SolidWorks CAD software and printed using an Original Prusa i3 MK3S+ printer.

### Drug Encapsulation in Macroporous ZIF‐8@Fe_3_O_4_ MMs

Before encapsulation, the MMs were dried overnight at 200 °C to remove any residual moisture from the pores. Subsequently, 0.1 mg of MMs (containing 2 000 000 MMs mL^−1^) was resuspended in 1 mL of 5‐FU, DOX, or a combination of both, at concentrations of 100, 500, and 1000 µm. The process began with 1 min of degassing in an ultrasonic bath, followed by 1 h at 50 °C and 500 rpm in a Thermomixer. The entire incubation process was conducted in the dark. After incubation, all samples were washed three times with water using a magnetic rack to retain the macroporous ZIF‐8@Fe_3_O_4_ MMs during the washing steps. For sequential incubation, the MMs were first incubated for 1 h with one drug, washed three times, incubated for another hour with the second drug, and then washed three times again.

### Drug Release Experiments

The pellet containing the drug‐loaded MMs was resuspended in water at 200 µL per initial 0.1 mg, yielding a concentration of 10 000 000 micromotors/mL. Fifty µL of the suspension, containing 500 000 MMs, was added to a microplate well and mixed with 50 µL of H_2_SO_4_ (≈0.1 mm) to obtain a final pH of 4. Experiments were conducted for 1, 12, 24, or 48 h at 37 °C in the dark, to simulate the conditions of a cell culture. This part was carried out under either static or magnetic stirring conditions (3.3 Hz). After incubation, the MMs were retained at the bottom of the well using a magnet, and the supernatant was transferred to a new microplate for reading under the same conditions as the calibration curve. A water blank was also read to subtract its value from the readings of each experimental condition. A calibration curve of 5‐FU and DOX concentration versus fluorescence was created to compare and quantify the drug release from ZIF‐8@Fe_3_O_4_ MMs. In the case of simultaneous incubation with 5‐FU and DOX, the fluorescence values were measured and the concentrations were calculated separately. This study was performed in triplicate. For acetate buffer experiments, MMs were mixed with 50 µL of acetate buffer (2 m) to achieve a final pH of 4.

For the calibration of 5‐FU, dilutions of 0, 25, 50, 75, 100, 125, 150, and 175 µm were prepared and fluorescence was measured with excitation at 265 nm and emission at 359 nm. For DOX, dilutions of 0, 0.25, 0.50, 0.75, 1, 1.25, 1.50, and 1.75 µm were measured with excitation at 480 nm and emission at 595 nm. For both drugs, 100 µL of each dilution was used, with measurements taken at a bandwidth of 10 nm, from a 7 mm top position and with a gain of 150. All measurements were performed at 37 °C to ensure the same conditions as for the drug release experiments. A water blank was also read to subtract its value from the concentration readings. This study was performed in triplicate. To study the mechanism of drug release, the fluorescence emission recorded at each release condition was converted to concentration using interpolation into the calibration plots.

### Drug Release Into Cell Cultures

Cytotoxicity assays were conducted at the Cell Culture Centre of the University of Alcalá, with the residual MMs removed. The results were obtained from three replicates of Caco‐2 cells incubated under various conditions and for different time periods. Before conducting the cytotoxicity assay, images of the cell state were captured using both optical and confocal microscopes at the Cell Culture Centre of the University of Alcalá.

### Statistical Analysis

The statistical significance between the experimental groups and the corresponding control experiments was assessed using t‐tests (α = 0.05) with Bonferroni correction. All statistical analyses were performed using OriginPro software for data manipulation and analysis.

## Conflict of Interest

The authors declare no conflict of interest.

## Supporting information



Supporting Information

Supplemental Video 1

Supplemental Video 2

Supplemental Video 3

Supplemental Video 4

Supplemental Video 5

Supplemental Video 6

## Data Availability

The data that support the findings of this study are available on request from the corresponding author. The data are not publicly available due to privacy or ethical restrictions.
