# Supplementary material for: Self‐Disassembling Macroporous Metal–Organic Framework‐Based Micromotors with Magnetically Controlled Motion for Sequential Drug Release
Source: Small Methods. 2025 Jun 3;9(10):2500724. doi: 10.1002/smtd.202500724 (PMC12536394; doi:10.1002/smtd.202500724)
Supplement: Supplementary file 1 — Supporting Information [file SMTD-9-2500724-s007.docx]

**SUPPORTING INFORMATION**

**Self-Disassembling Macroporous Metal-Organic Framework based Micromotors with Magnetically Controlled Motion for Sequential Drug Release**

Javier Bujalance-Fernández,^[a]^ Víctor de la Asunción-Nadal,^[a]^ Beatriz Jurado-Sánchez,*^[a,b]^ and Alberto Escarpa*^[a,b]^

[a] Department of Analytical Chemistry, Physical Chemistry, and Chemical Engineering, Universidad de Alcala, Alcala de Henares, E-28802 Madrid, Spain, E-mail: beatriz.jurado@uah.es; alberto.escarpa@uah.es

[b] Chemical Research Institute “Andres M. Del Río”, Alcala de Henares, E-28802 Madrid, Spain

**Table of Contents**

Results and Discussions ---………………………………………………………………………………………………………………………..2

References…………………………………………………………………………………………………………………………………………12

**
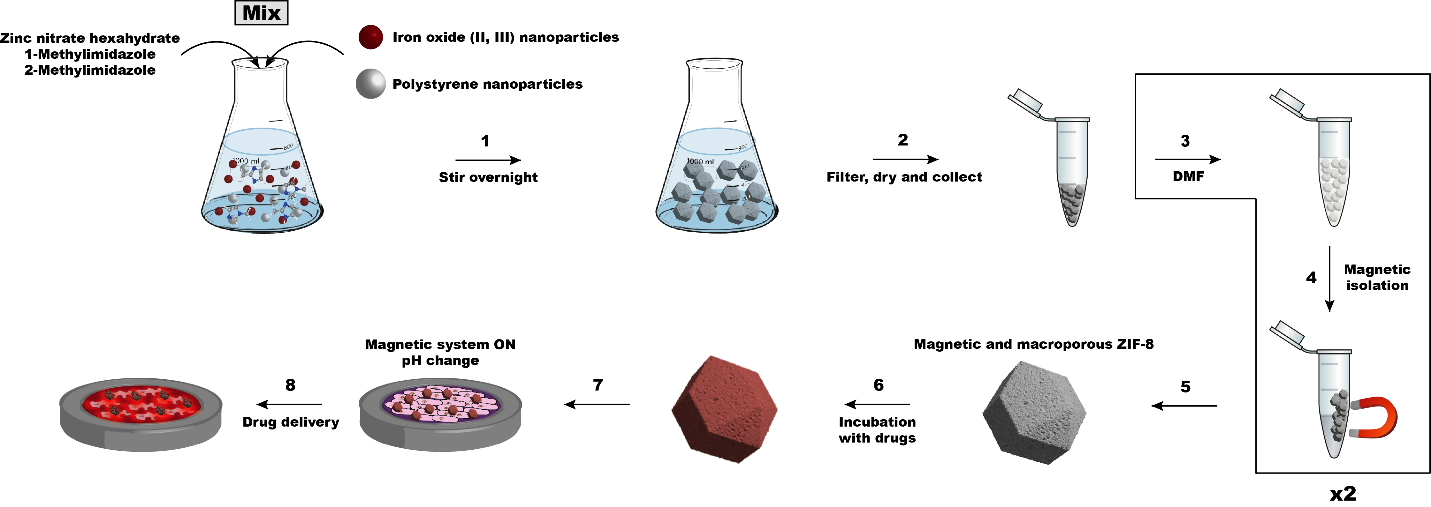
**

**Figure S1.** Schematic of the synthesis of macroporous ZIF-8@Fe_3_O_4_ MMs. 1: synthesis; 2: filter, dry and collect; 3, 4: polystyrene degradation; 5: isolation; 6: incubation with drugs; 7: ZIF-8@Fe_3_O_4_ solution drop in Caco-2 cell culture and 8: pH change and activation of a magnetic system for drug delivery.


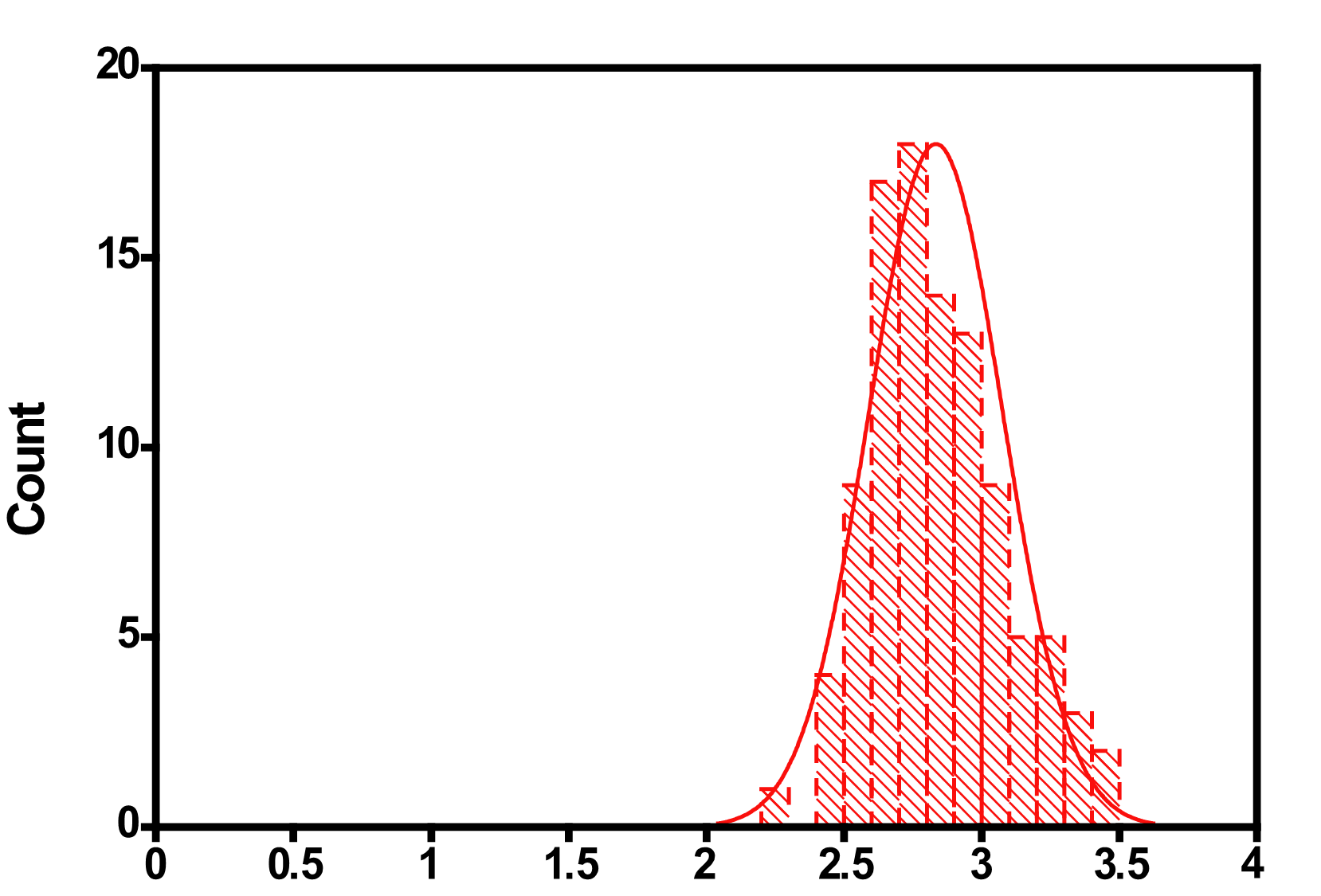


**Figure S2.** Plot showing the size distribution of the ZIF-8@Fe_3_O_4_ MMs.


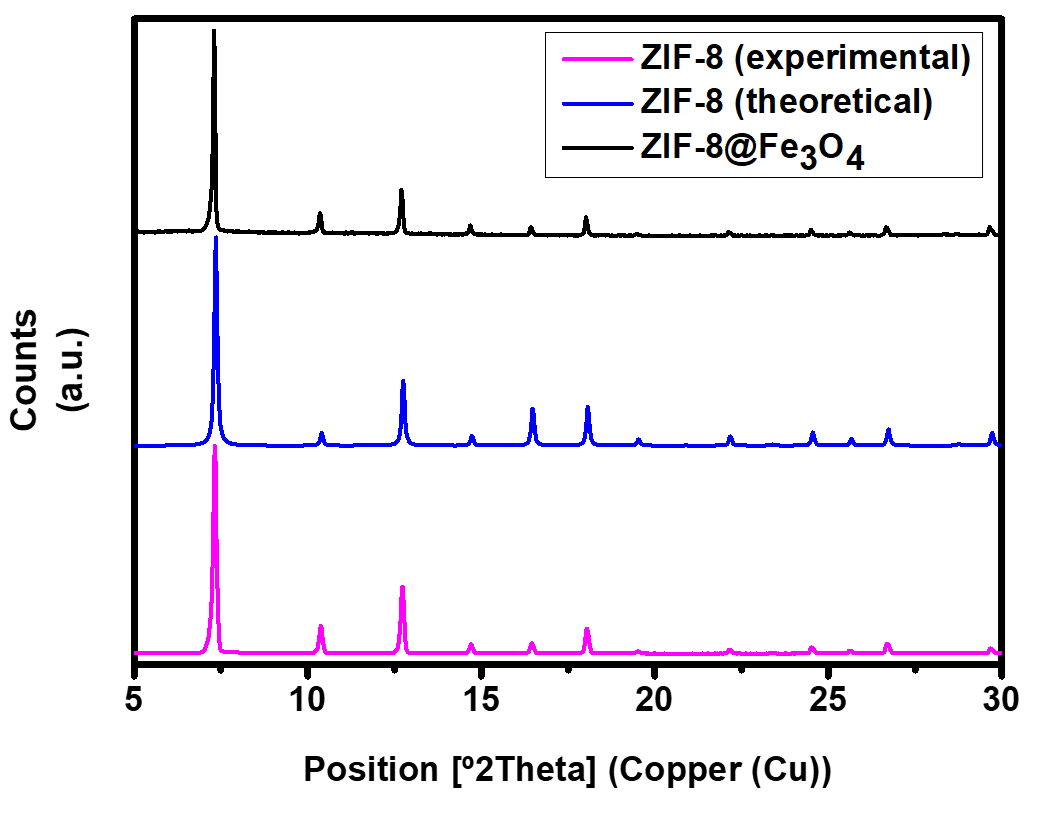


**Figure S3.** Experimental XRD spectrum of ZIF-8 and ZIF-8@Fe_3_O_4_ MMs and theoretical XRD spectrum of ZIF-8.


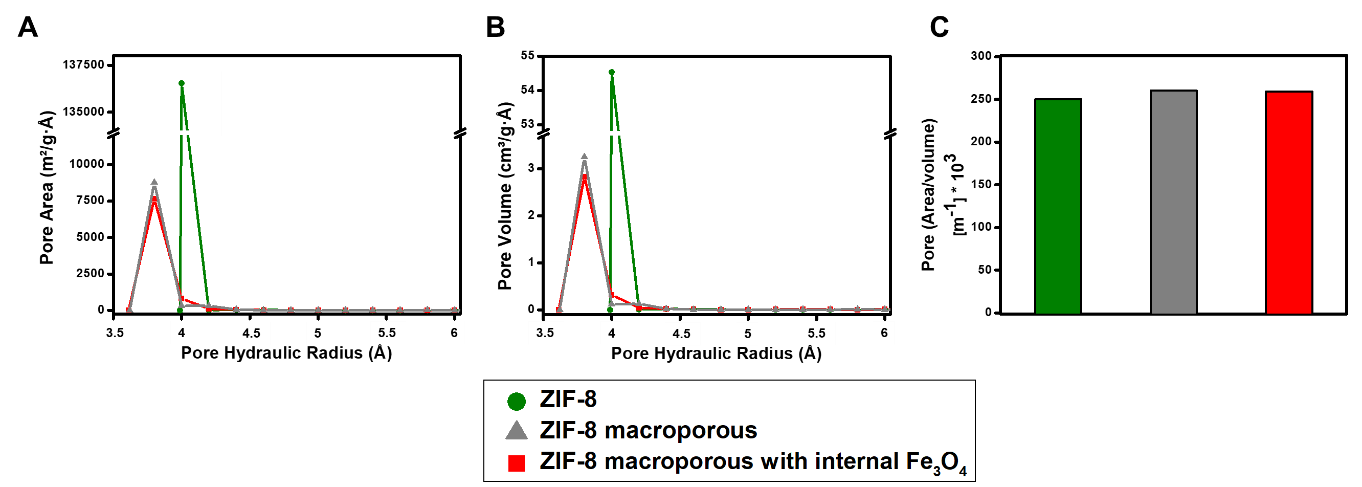


**Figure S4.** Micropore analysis evaluation of the pore size of the MMs. (A) pore area, (B) pore volume and (C) area/volume ratio.


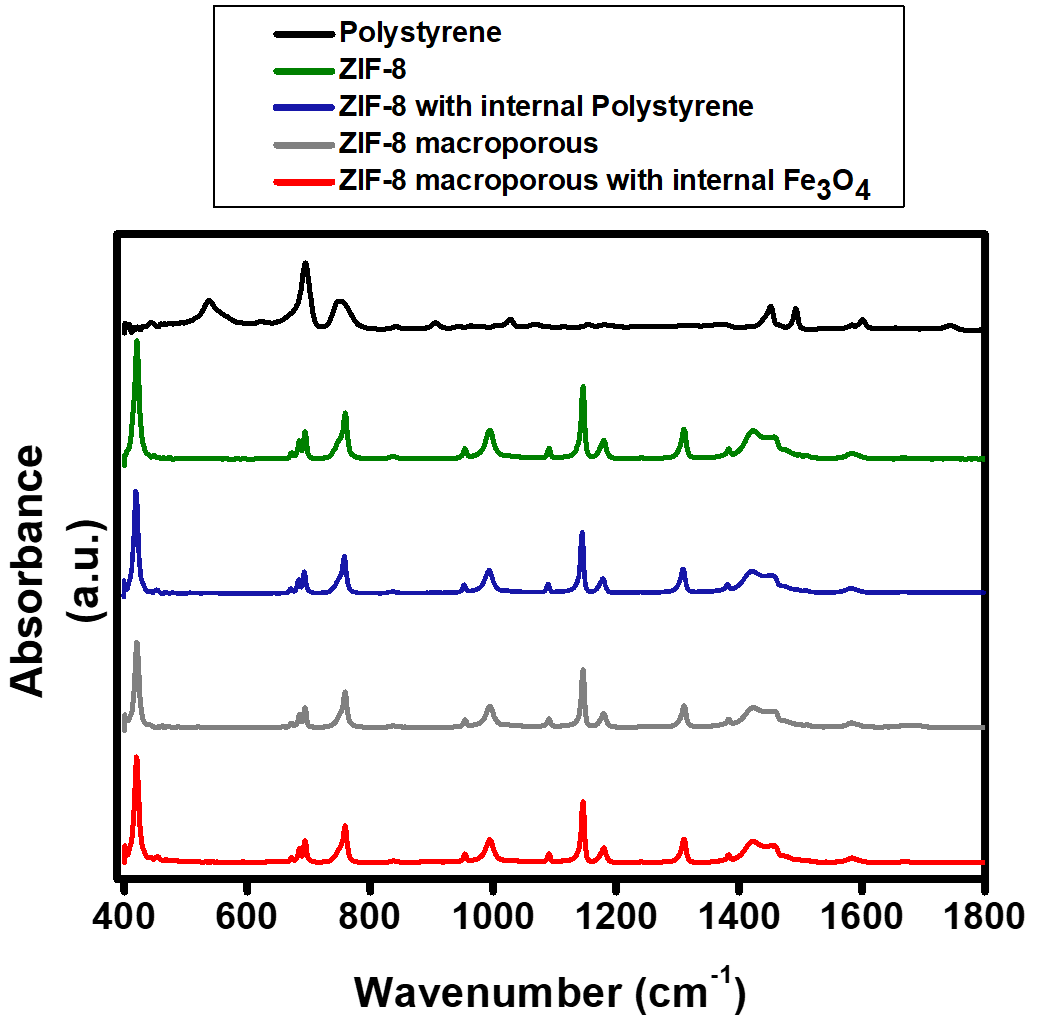


**Figure S5.** ATR-IR characterization of the MMs.


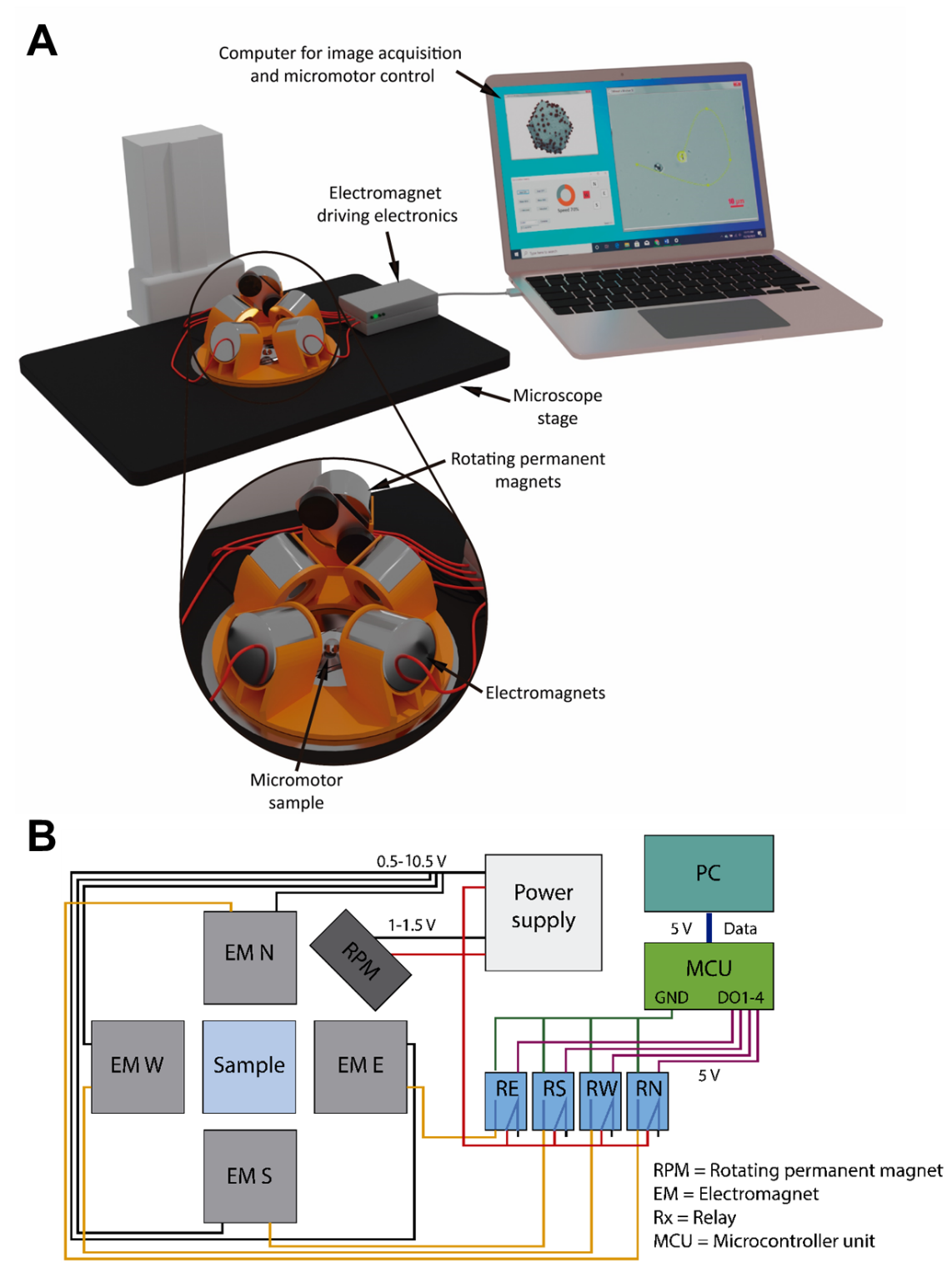


**Figure S6. (A)** Schematic of the 3D-printed electromagnetic system used to control motion. (B) Wiring schematic of the electronic control system of the electromagnets and rotating permanent magnets.

**
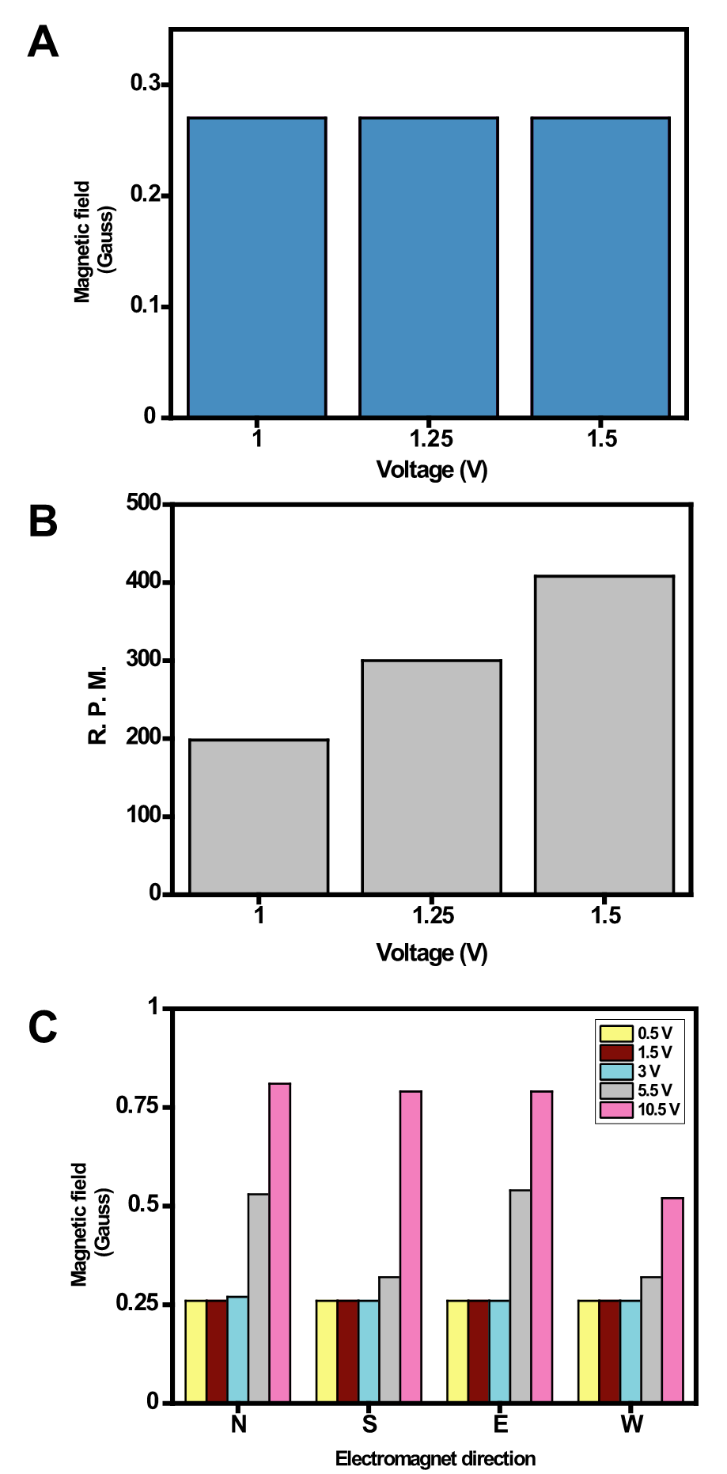
**

**Figure S7.** (A) Magnetic fields at the drop by the rotating permanent magnets at different voltage. (B) Revolutions per minute of the rotating permanent magnets at different voltage. (C) Magnetic fields at the drop using the electromagnets at different voltages (n=3 in all experiments)

**Figure S8.** A) Speed measurements of magnetic macroporous ZIF-8@Fe_3_O_4_ MMs in different media. B) SEM images after 24 h incubation of the MMs in acetate buffer (a), gastric acid (b) and serum (c). Error bars represent the mean values ± standard deviation (n=3). Scale bars, 5 µm.


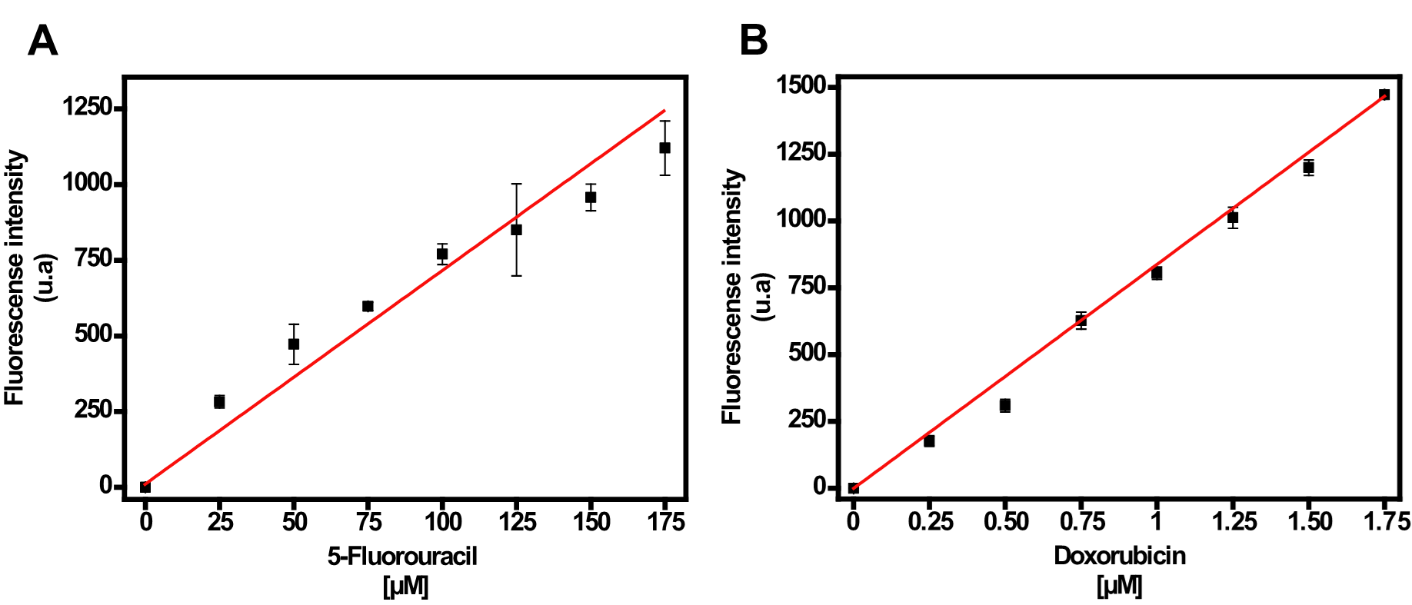


**Figure S9.** Corresponding calibration plots of fluorescence values of different concentrations of (A) 5-FU and (B) DOX. Fluorescence values were plotted by subtracting the fluorescence values of water from drug values. Error bars represent the mean values ± standard deviation (n=3).


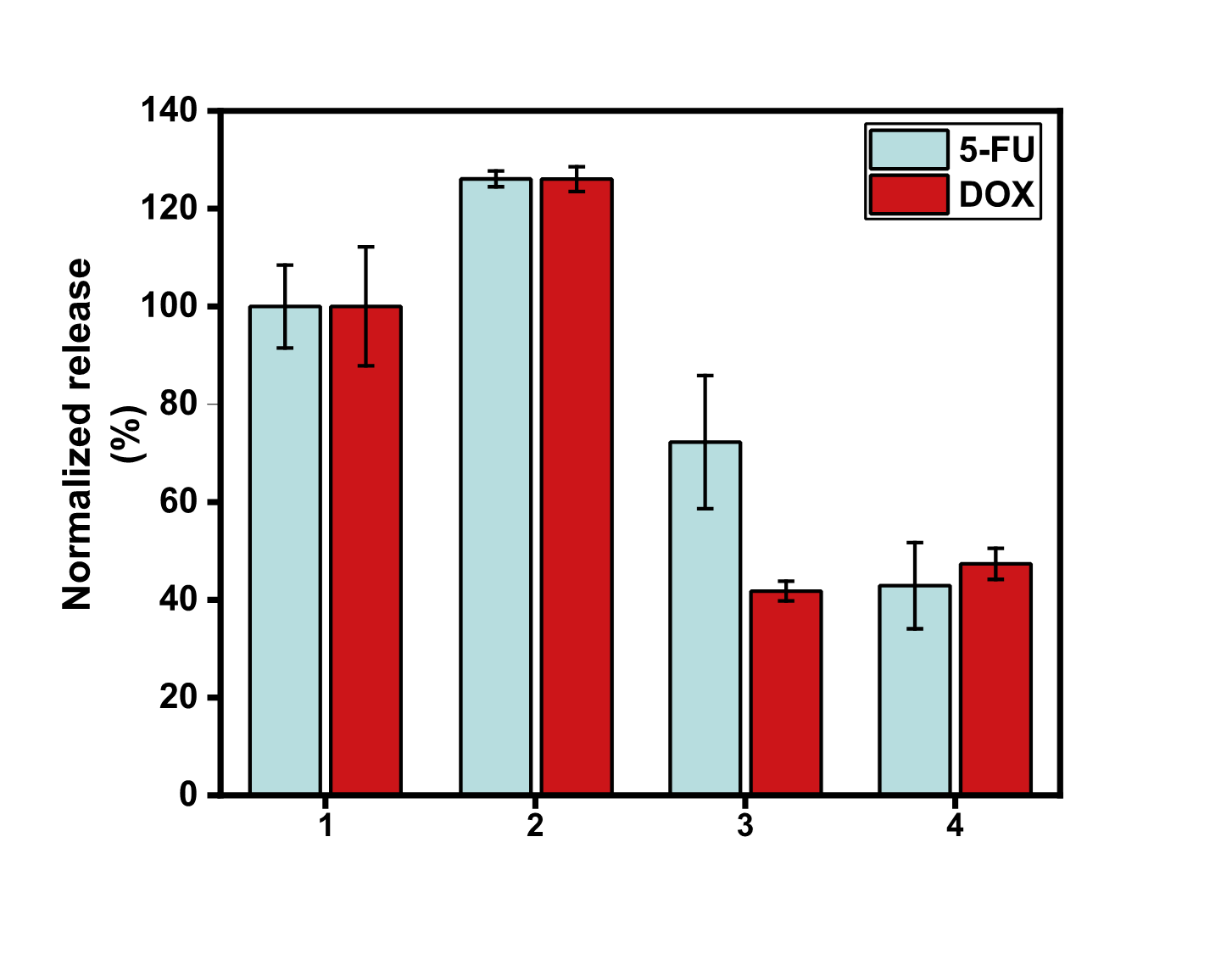


**Figure S10.** Drug loading (in terms of released concentration) into the (1) magnetic macroporous ZIF-8@Fe_3_O_4_ MMs at pH 4 previously incubated with a mixture containing 1 mM concentration of 5-FU and DOX; (2) magnetic macroporous ZIF-8@Fe_3_O_4_ MMs incubated first with 1 mM 5-FU followed by 1 mM of DOX; (3) magnetic macroporous ZIF-8@Fe_3_O_4_ MMs incubated first with 1 mM DOX followed by 1 mM of 5-FU and (4) microporous ZIF-8@Fe_3_O_4_ incubated with a mixture containing 1 mM concentration of 5-FU and DOX. Error bars represent the mean values ± standard deviation (n=3).

**
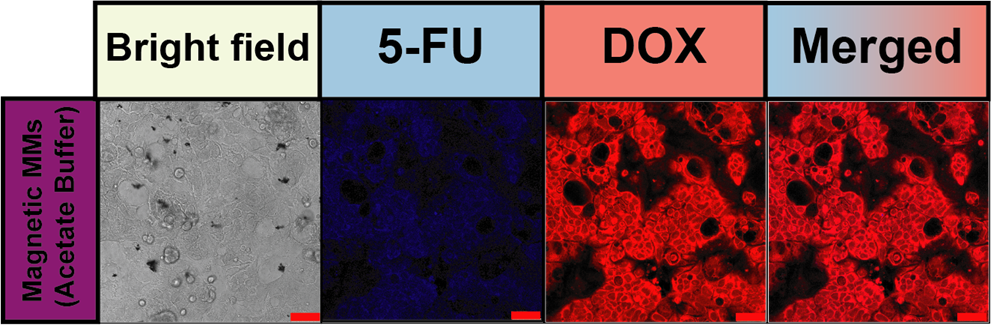
**

**Figure S11.** Confocal images of Caco-2 cells after 24 hours in acetate buffer (pH 4.0) using MM under magnetic conditions previously incubated with 1000 µM of 5-FU and 1000 µM of DOX simultaneously. Scale bars, 50 μm.


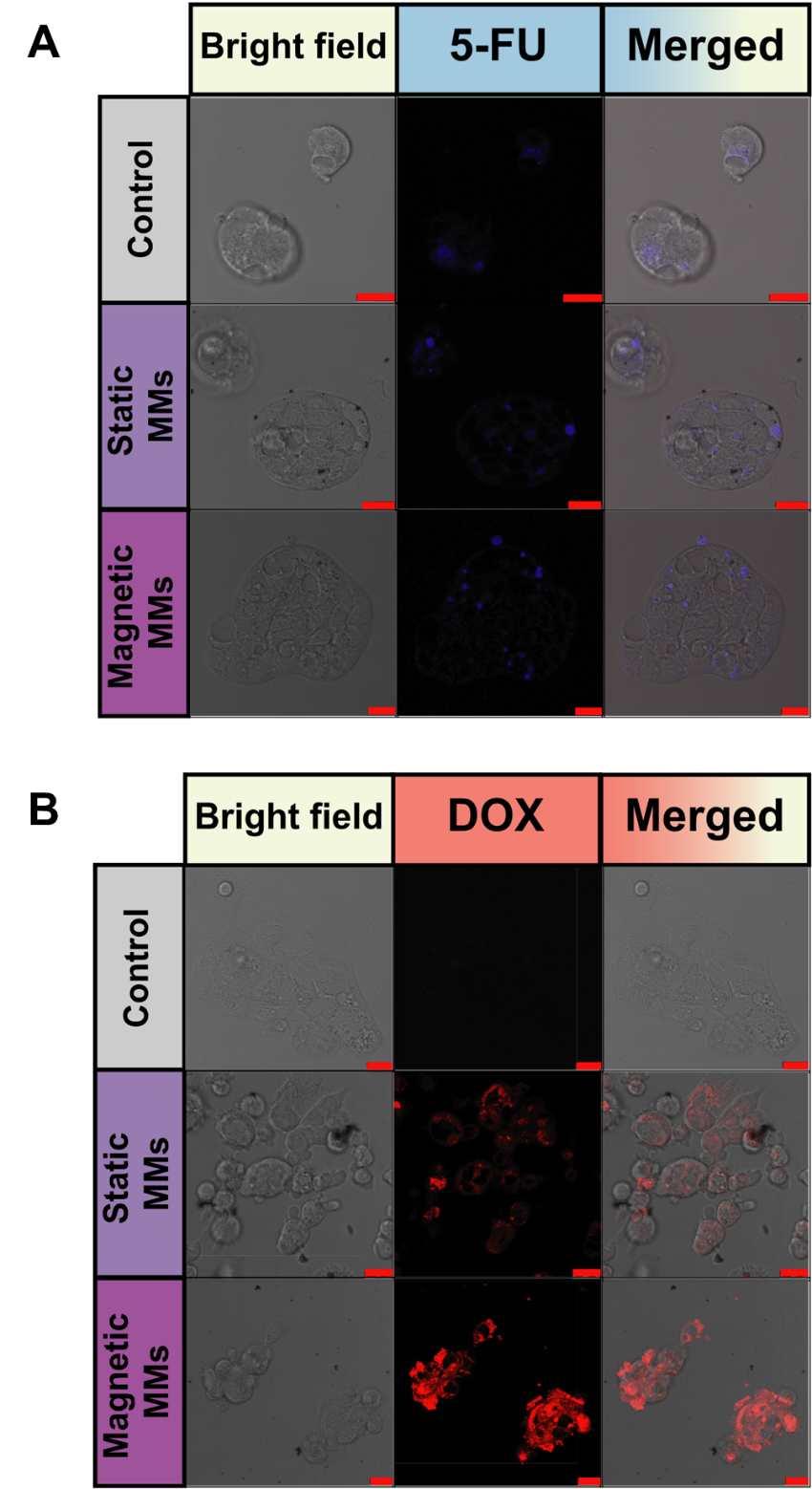


**Figure S12.** Confocal images of Caco-2 cells after 24 hours in sulfuric acid at pH 4 of untreated sample (Control) and with MMs under static or magnetic conditions previously incubated with (A) 1000 µM of 5-FU or with (B) 1000 µM of DOX. Scale bars, 25 μm.

**
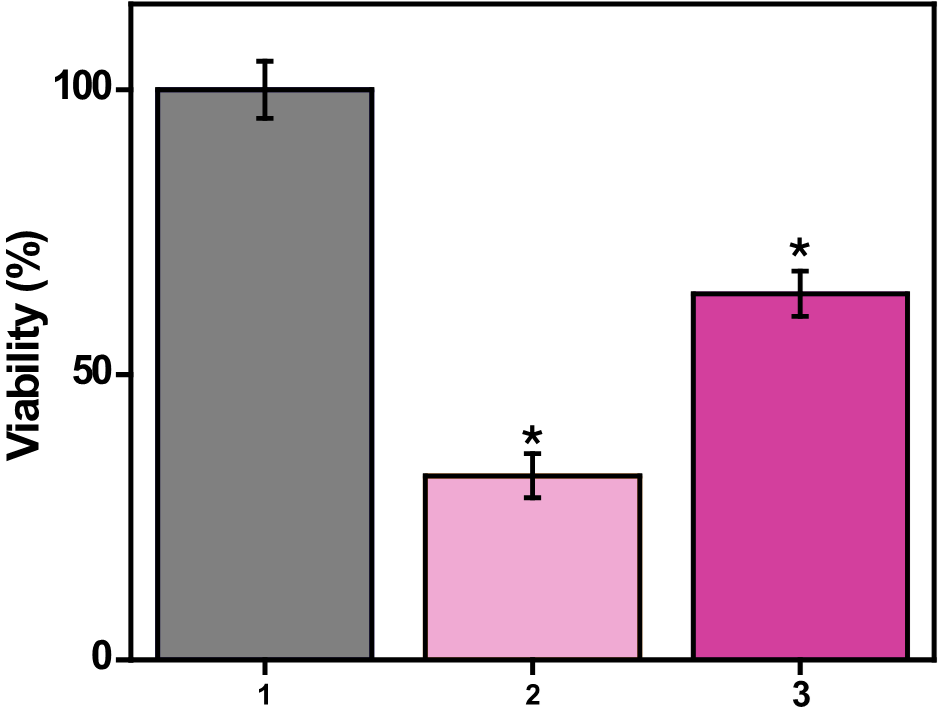
**

**Figure S13.** Corresponding MTT assays (n=3). Conditions: (1) pH 4 control, (2) magnetic macroporous ZIF-8@Fe_3_O_4_ MMs incubated first with 1 mM 5-FU followed by 1 mM of DOX and (3) magnetic macroporous ZIF-8@Fe_3_O_4_ MMs incubated first with 1 mM DOX followed by 1 mM of 5-FU. 500,000 MMs/treatment. Error bars represent the mean values ± standard deviation (n=3). ***** Statistically significant difference between study and control conditions (α = 0,05).

**Figure S14.** SEM images showing the interaction of the Caco-2 cell with the macroporous ZIF-8@Fe_3_O_4_ MMs after 24 incubation and drug release. Scale bars, 5 μm.

**Table S1.** MOFs based MMs for drug delivery comparison

| **Composition** | **Drug (released concentration)** | **Drug released** | **Cell model** | **Release time** | **Speed (µm/s)** | **Remarks** | **Ref.** |
| --- | --- | --- | --- | --- | --- | --- | --- |
| ZIF-L catalase catalytic MMs | 5-FU | - | MCF-7 | 24 h | 5170 (2 % H_2_O_2_) | -Biocatalytic which releases bubbles that are cell-unfriendly  -Unmeasured drug release  -Uncontrolled movement | [1] |
| ZIF-L succinylated β-lactoglobulin@catalase catalytic MMs | DOX (1840 µM) | 10 µM | HeLa | 48 h | - | -Biocatalytic  -pH response  -Uncontrolled movement | [2] |
| ZIF-67/Fe_3_O_4_ catalytic MMs | DOX  (920-7360 µM) | - | - | - | 51.8 (6 % H_2_O_2_) | -Biocatalytic which releases bubbles that are cell-unfriendly  -Unmeasured drug release  -Magnetic field control  -*In vitro* study not performed | [3] |
| CuS-ZIF-8 NIR propelled MMs | DOX (368 µM) | - | MCF-7 | 24 h | - | -Near-infrared control  -pH response  -Unmeasured drug release  -Intracellular release  -*In vivo* study | [4] |
| Gelatin methacryloyl helices@Fe@ZIF-8 magnetic MMs | DOX (92 µM) | 1.8 µM | MDA-MB-231 | 48 h | - | -Magnetic field control  -pH response | [5] |
| ZIF-8@Fe magnetic MMs | DOX (86 µM) | 80 µM | MCF-7 | 48 h | - | -Magnetic field control  -pH response  -*In vivo* study | [6] |
| ZIF-8@Fe_3_O_4_ magnetic MMs | 5-FU (1000 µM)  DOX (1000 µM) | 5-FU  (140 µM)  DOX (1 µM) | Caco-2 | 24 h | 9.7-13.8  (total speed)  1-1.4 (linear speed) | -Tunable pore size  -Adjustable drug loading  -pH response  -Magnetic field control by software  -First MOF based MM with a simultaneous drug release | This work |

**References**

1. Z. Guo, T. Wang, A. Rawal, J. Hou, Z. Cao, H. Zhang, J. Xu, Z. Gu, V. Chen and K. Liang, Biocatalytic Self-Propelled Submarine-Like Metal-Organic Framework Microparticles with pH-Triggered Buoyancy Control for Directional Vertical Motion, *Mat. Tod.*, **2019**, *28*, 10-16.
2. S. Gao, J. Hou, J. Zeng, J. J. Richardson, Z. Gu, X. Gao, D. Li, M. Gao, D.-W. Wang, P. Chen, V. Chen, K. Liang, D. Zhao and B. Kong, Superassembled Biocatalytic Porous Framework Micromotors with Reversible and Sensitive pH-Speed Regulation at Ultralow Physiological H_2_O_2_ Concentration, *Adv. Function. Mater.*, **2019**, *29*, 1808900.
3. L. Wang, H. Zhu, Y. Shi, Y. Ge, X. Feng, R. Liu, Y. Li, Y. Ma and L. Wang, Novel Catalytic Micromotor of Porous Zeolitic Imidazolate Framework-67 for Precise Drug Delivery, *Nanoscale*, **2018**, *10*, 11384-11391.
4. C.-G. Liu, C.-P. Fu, Y.-H. Shi, J. Zhong, H.-X. Tang, J.-T. Zhang, R. Kumar Kankala, S.-B. Wang and A.-Z. Chen, Dual-Responsive Nanomotors for Deep Tumor Penetration and Subcellular Arrangement, *Mat. Design*, **2022**, *222*, 111039.
5. A. Terzopoulou, X. Wang, X.-Z. Chen, M. Palacios-Corella, C. Pujante, J. Herrero-Martín, X.-H. Qin, J. Sort, A. J. deMello, B. J. Nelson, J. Puigmartí-Luis and S. Pané, Biodegradable Metal–Organic Framework-Based Microrobots (MOFBOTs), *Adv. Health. Mater.*, **2020**, *9*, 2001031.
6. Q. Cao, Y. Zhang, Y. Tang, C. Wu, J. Wang and D. Li, MOF-based magnetic microrobot swarms for pH-responsive targeted drug delivery, *Sci. China Chem.*, **2024**, *67*, 1216-1223.
